# Novel A-π-D-A organic dyes for better photovoltaic performance†

**DOI:** 10.1039/d4ra05341a

**Published:** 2024-09-16

**Authors:** Krupa Elsa Roys, S. L. Manju, Mohamed Siddiq, Anandan Sambandam

**Affiliations:** a Department of Chemistry, School of Advanced Sciences, Vellore Institute of Technology Vellore Tamil Nadu 632014 India slmanju@vit.ac.in; b Nanomaterials and Solar Energy Conversion Laboratory, Department of Chemistry, National Institute of Technology Trichy 620015 India sanand@nitt.edu

## Abstract

In this article, we report two indole-based metal-free organic dyes In-T-2C (3-(5-(3-((2-carboxy-2-cyanovinyl)-1-pentyl-1*H*-indol-5-yl)thiophen-2-yl)-2-isocyanoacrylic acid) and In-B-C (2-cyano-3-(5-(4-cyanophenyl)-1-pentyl-1*H*-indol-3-yl)acrylic acid) with A-π-D-A architecture. The molecular structures of metal-free indole-based A-π-D-A organic dyes were elucidated using FT-IR, NMR, HRMS and single-crystal X-ray diffraction techniques. The present investigation examined the features of the synthesized dyes employing photophysical attributes, electrochemical traits and theoretical studies were executed to acquire a detailed comprehension of the geometry, electronic structure and absorption spectra of the synthesized dyes using density functional theory (DFT) and time-dependent density functional theory (TD-DFT). Additionally, dye-sensitized solar cells (DSSCs) were fabricated using newly synthesized dyes and examined their photovoltaic activity. Electrochemical impedance analysis (EIS) was performed to recognize the interfacial charge transfer in the DSSCs. The In-T-2C dye-based DSSC device exhibited an uppermost fill factor (FF) of 0.63, resulting in the uppermost open-circuit voltage (*V*_OC_) of 540.2 mV and highest efficiency (*η*) of 4.12% due to the highest short-circuit current density (*J*_SC_) of 12.1 mA cm^−2^ compared to the In-B-C dye (*V*_OC_ = 497 mV, *J*_SC_ = 1.07, FF = 0.70, *η* = 0.38%).

## Abstract 2

Novel A-π-D-A type indole-based metal-free organic dyes have been designed and synthesized for dye-sensitized solar cells (DSSCs). In-T-2C dye-based DSSC device exhibited the highest efficiency (*η*) of 4.12% compared to In-B-C dye (*η* = 0.38%).

## Graphical abstract

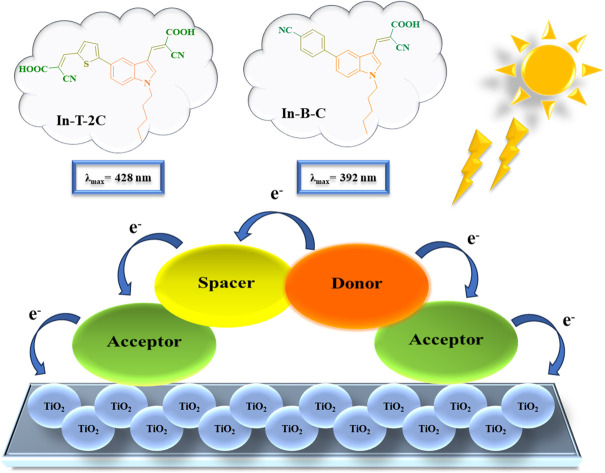

## 1 Introduction

The desire for energy production from renewable sources has escalated in recent decades, which has resulted in the evolution of advanced technologies like photovoltaics.^1^ For half a century, photovoltaic systems based on crystalline silicon (Si) have influenced the field of solar energy conversion due to their various advantageous characteristics, including superior efficiency as well as good stability under varying weather conditions. However, researchers switched from traditional photovoltaic technologies to third-generation technologies, including perovskite solar cells (PSCs) and dye-sensitized solar cells (DSSCs),^2,3^ and organic solar cells (OSCs) due to certain limitations such as high cost, restricted transportability, and low photovoltaic performance in low light intensities.^4^ Additionally, luminescent solar concentrators (LSCs) have been developed to address the limitations of optical concentrators, like the requirement for cooling mechanisms to disperse the excess heat from unconverted energy and rotation mechanisms to enable the concentrator to track the motion of the sun.^5,6^ Since Michael Gratzel implemented dye-sensitized solar cells (DSSCs) in 1991, there has been a lot of interest in their potential to convert solar energy into electrical energy using an effective solar cell.^7,8^ The four primary elements included in DSSCs are the photoanode, the electrolyte, the counter electrode, and the sensitizer (dye). The sensitizer is a crucial factor of DSSCs because they have a direct impact on the solar cell's capacity to absorb light and convert it into electrical energy, as well as determine the cell's power conversion efficiency. The three distinct components that comprise the sensitizer moiety are the donor (D), acceptor (A) and spacer (π), which facilitate an electron push–pull process.^9^ Strong charge-transfer absorption bands emerge from the electronic interaction across the donor (D) and acceptor (A) moiety *via* the spacer units, which accumulate solar radiation for the conversion of photons to electrons.^10^ As the enhanced photophysical and electrochemical features of Ru(ii) complexes make them outstanding sensitizers for DSSCs, the necessity and usage of rare noble metals, cost of manufacture, hazards to the environment, and purification struggles have restricted their growth.^11^ Metal-free organic dyes have multiple advantages over Ru(ii) complexes like N719 and NCSU-10 including accessibility, geometric flexibility, absorption across the near-IR and UV-visible regions, outstanding light harvesting features, and inexpensive synthesis methods. Researchers designed and nurtured various metal-free organic dyes for DSSC based on donor (D), acceptor (A) and spacer (π) structured manner, such as D-π-D-π-A type phenothiazine-imidazole based sensitizers,^12^ D-D-π-A type indole-based sensitizers,^13–15^ D-D-π-A type imidazole-based sensitizers,^16,17^ D-π-A type indeno[1,2-*b*]indole based sensitizers,^18^ A-π-D-π-A-π-A type carbazole-based sensitizers,^19^ D-π-A type thieno[2,3-*b*]indole-based sensitizers,^20^ D-A-π-A type 2-phenothiazine-phenylamine-based sensitizers,^21^ D-D-π-A type indolo[2,3-*b*]quinoxaline based sensitizers,^22^*etc.*

Researchers have become fascinated by heterocyclic moieties like indoles as a result of their robust charge transfer, adaptive synthetic properties, and electron-rich framework.^18,23,24^ Along with the donor unit, the spacer unit as well as the acceptor moiety in the sensitizer plays a crucial part in the functioning of DSSC. Rhodanine-3-acetic acid and cyanoacetic acid are commonly utilized as acceptor units because of their effective ability to capture electrons and anchor on the surface of TiO_2_.^15^ Thiophene, furan and benzene, *etc* are frequently used as spacer moieties which help in the delocalization of electrons from the donor side to the acceptor unit.^25^

**Fig. 1:**
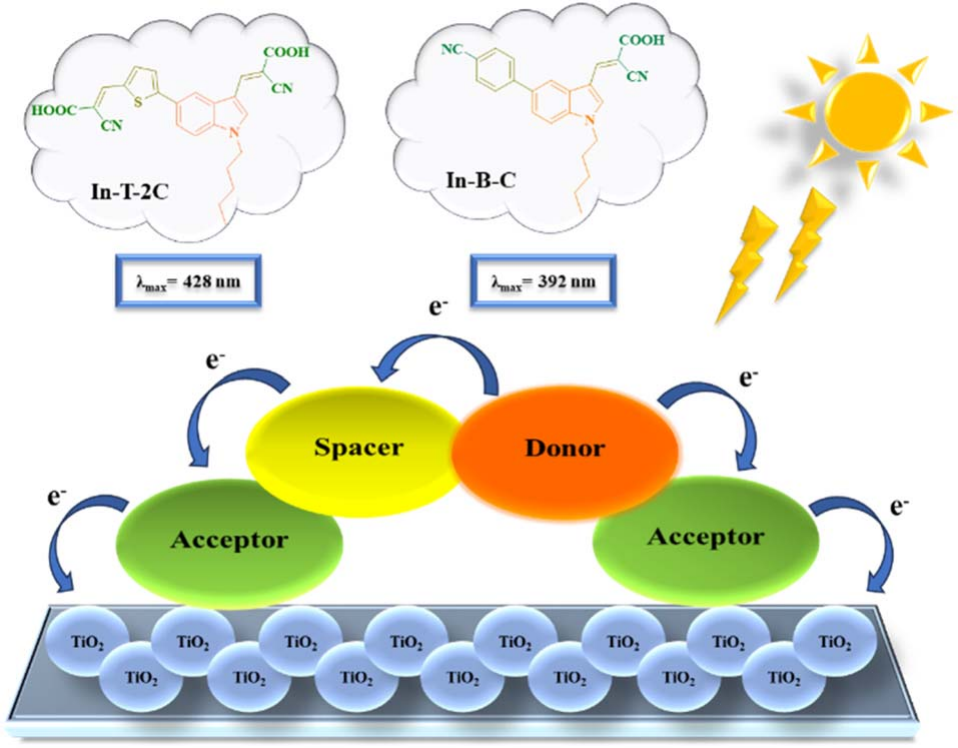
Schematic illustration of A-π-D-A structured indole-based dyes.

Accordingly, we have designed and developed unique A-π-D-A structured dyes based on indole units. The synthesized dyes were thoroughly characterized using FT-IR, ^1^H NMR, ^13^C NMR, HRMS and single-crystal X-ray diffraction techniques. Moreover, photophysical features and electrochemical traits were examined. Using the B3LYP/6-31G (d, p) basis set in Gaussian 16, density functional theory (DFT) and time-dependent density functional theory (TD-DFT) were executed to acquire a detailed comprehension of the geometry, electronic structure and absorption spectra of the newly synthesized dyes.^26–29^ Additionally, DSSCs were fabricated by using the synthesized dyes and examined their photovoltaic activity. Electrochemical impedance analysis (EIS) was performed to recognize the interfacial charge transfer as well as recombination in the dye-sensitized solar cells (DSSCs).^30^Fig. 1 displays the schematic illustration of unique A-π-D-A structured indole-based dyes.

## 2 Results and discussion

### 2.1. Synthesis

**Scheme 1:**
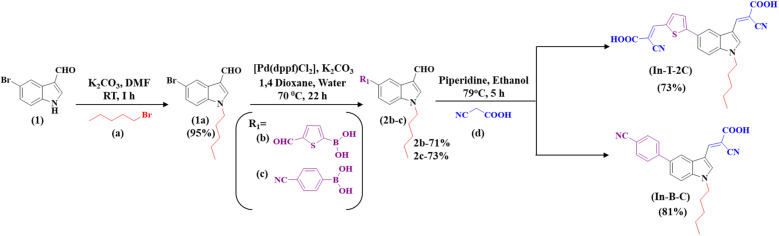
Synthetic approach for the A-π-D-A structured indole-based dyes.

**Fig. 2:**
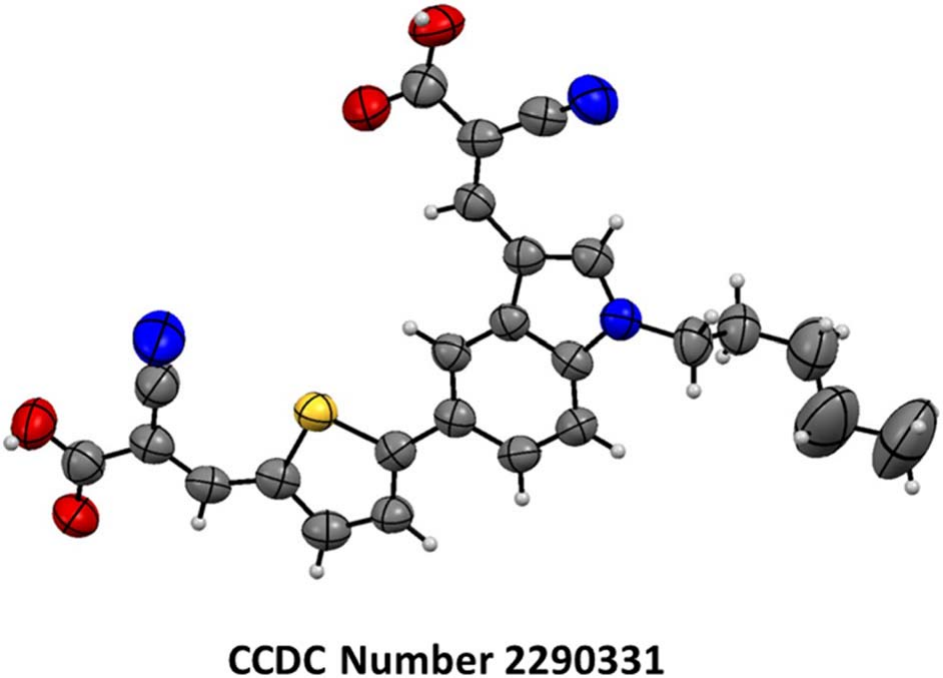
The ORTEP diagram and CCDC number of In-T-2C dye.

Scheme 1 demonstrates the synthetic pathway leading to the new metal-free indole-based dyes. Using potassium carbonate as a base in a dimethylformamide (DMF) solution at room temperature for two hours, 5-bromoindole-3-carboxaldehyde (1) was *N*-alkylated using 1-bromopentane (a) to yield 5-bromo-1-pentyl-1*H*-indole-3-carbaldehyde (1a) compound in a 95% yield. Compound 1a was subjected to a Suzuki coupling reaction in the presence of 5-formyl-2-thienylboronic acid (b), potassium carbonate, and [1,1′-Bis(diphenylphosphino)ferrocene] dichloropalladium(ii) [Pd(dppf)Cl_2_] in a mixture of 1,4-dioxane and water in a 3 : 1 ratio. This reaction resulted in the formation of a 5-(5-formylthiophen-2-yl)-1-pentyl-1*H*-indole-3-carbaldehyde (2b) compound in a 71% yield. The Knoevenagel reaction was utilized to synthesize 3-(5-(3-((2-carboxy-2-cyanovinyl)-1-pentyl-1*H*-indol-5-yl)thiophen-2-yl)-2-isocyanoacrylic acid) (In-T-2C) compound from 2b using cyanoacetic acid and piperidine as the base, resulting in a 73% yield. Likewise, compound 1a was utilized to obtain the 4-(3-formyl-1-pentyl-1*H*-indol-5-yl)benzonitrile (2c) (73%) through Suzuki coupling reaction in the presence of potassium carbonate and [1,1′-Bis(diphenylphosphino)ferrocene] dichloropalladium(ii) [Pd(dppf)Cl_2_] in a mixture of 1,4-dioxane and water in a 3 : 1 ratio. Compound 2c was subjected to a Knoevenagel reaction with cyanoacetic acid and piperidine as the base, resulting in the formation of 2-cyano-3-(5-(4-cyanophenyl)-1-pentyl-1*H*-indol-3-yl)acrylic acid (In-B-C) with 81% yield. The structural elucidation of the In-T-2C and In-B-C is shown using its characterization details. The product In-T-2C was recrystallized, the ORTEP diagram and CCDC number are provided in Fig. 2.

### 2.2. Photophysical properties

**Fig. 3:**
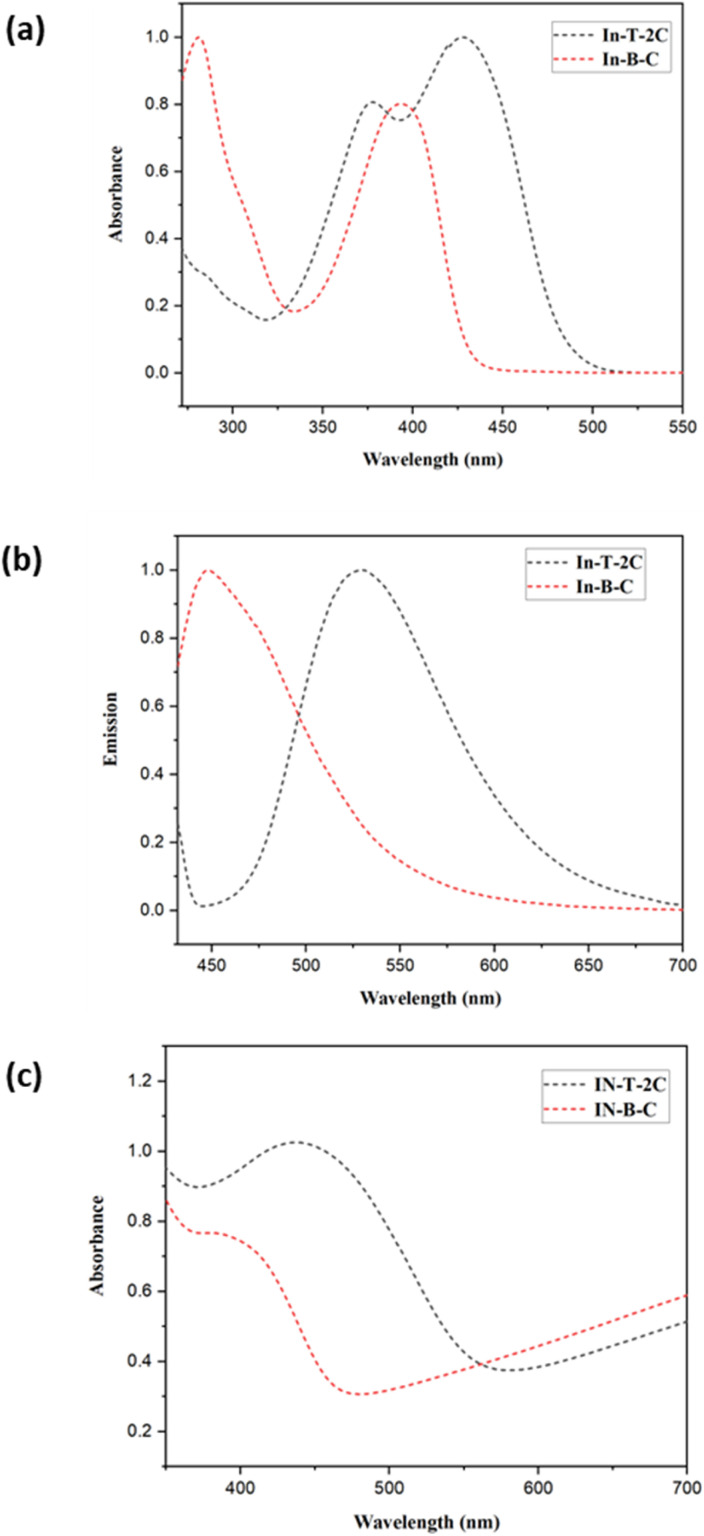
(a) Normalized UV-Visible spectra and (b) normalized emission spectra of In-T-2C and In-B-C in DMSO (1 × 10^− 5^ M), (c) UV-DRS spectra of synthesized compounds on TiO_2_.

The UV-visible and emission spectra of the A-π-D-A structured compounds were investigated and evaluated in dimethyl sulfoxide (DMSO) at a concentration of 1 × 10^−5^ M to study their photophysical attributes. The relevant optical properties as well as the UV-visible and emission spectra of unique A-π-D-A structured dyes are provided in Table 1 and Fig. 3a and b. The novel In-T-2C and In-B-C dyes, depicted two significant absorption bands in the region of 281–377 nm and 392–428 nm. The peaks corresponding to the higher energy region of 281–377 nm are because of the π–π* electronic excitations, which are confined within the indole donor.^31^ The peaks corresponding to the lower energy exhibited among 392 and 428 nm are responsible for the intramolecular charge transfer (ICT) among the donor and the acceptor moieties.^17^ The uppermost absorption band (*λ*_max_) of In-T-2C is detected at 428 nm and for In-B-C the topmost absorption peak (*λ*_max_) is located at 392 nm. Also, it discovered that the absorption band responsible for the intramolecular charge transfer (ICT) in the In-T-2C compound is red-shifted than the In-B-C compound, because of the presence of thiophene moiety as a linker in In-T-2C which improves the electron delocalization than the benzene moiety existing in In-B-C compound^25^ and also the presence of two cyanoacetic acid units in In-T-2C compound as acceptor moieties helps in effective electron extraction from indole donor.^32^ Upon comparison to our previous research, the A-π-D-A structured In-T-2C compound disclosed higher absorption than the reported D-π-A and D-D-π-A indole-based metal-free compounds.^29,33^

**Table 1:** Optical characteristics of dyes In-T-2C and In-B-C in DMSO (1 × 10^−5^ M)

In DMSO	Absorption				Emission	Stokes shift	DRS optical energy gap
	*λ*_max_ (nm) (1)	*λ*_max_ (nm) (2)	*ε* (10^4^ M^−1^ cm^−1^) (2)	*λ*_max_ (nm) (TiO_2_)	*λ*_max_ (nm)	SS (cm^−1^)	*E*_g_ (eV)
In-T-2C	377	428	9.857	439	530	4497	2.00
In-B-C	281	392	9.870	387	447	3139	2.50

Fig. 3c demonstrates the UV-DRS spectra of the synthesized compounds on the TiO_2_ surface. The In-T-2C and In-B-C dyes on the TiO_2_ surface reveal maximum absorption peaks at 439 nm and 387 nm, respectively. The absorption maxima for In-T-2C dye were red-shifted and In-B-C dye was blue-shifted compared to their absorption maxima in DMSO. Deprotonation and the aggregation effects such as H-type and J-type aggregation are the primary factors influencing the change in the UV-Vis absorption spectra.^34^ H aggregations mostly arise from the hypsochromic or blue shift of the dye anchored on TiO_2_, while J aggregations typically result from the bathochromic or red shift.^35^ Accordingly, the red shift phenomenon of In-T-2C dye suggests that there is a certain degree of J-type aggregation on the TiO_2_ films. The interaction among the carboxylate group of the sensitizer and the TiO_2_ semiconductor is responsible for the generation of J-aggregated molecules.^22^ Also, the blue shift phenomenon of In-B-C dye is due to the H aggregation.^19^ Comparatively, In-T-2C shows a red shift and a broader absorption peak, which indicates that In-T-2C has a robust light harvesting ability on the TiO_2_ surface.^36^

The molar extinction coefficient values (*ε*, M^−1^ cm^−1^) of In-T-2C and In-B-C were assessed using the Beer–Lambert law. The molar extinction coefficient values of these dyes are astonishingly high at peaks responsible for the intramolecular charge transfer (ICT), for In-T-2C and In-B-C the molar extinction coefficient values are 9.857 × 10^4^ and 9.870 × 10^4^ M^−1^ cm^−1^, respectively. Multiple elements like extended structural conjugation, electronic transitions, donor–acceptor nature, solvent effect, and intermolecular interactions influenced the absorptivity of the compound.^37^ When compared to the reported simple organic dyes such as D-π-A type indole-based compounds^33^ demonstrated the molar extinction coefficient values between 5.436 × 10^4^ to 9.424 × 10^4^ M^−1^ cm^−1^, D-D-π-A structured indole-based dyes reported the molar extinction coefficient values between 4.907 × 10^4^ to 7.327 × 10^4^ M^−1^ cm^−1^, azo thiazole organic dyes^38^ demonstrated the molar extinction coefficient values between 5.12 × 10^4^ to 10.75 × 10^4^ M^−1^ cm^−1^, 3-(1-hexyl-1*H*-indol-3-yl)-2-(thiophen-2-yl) acrylonitrile based organic dyes^15^ display molar extinction coefficient values between 5.6 × 10^4^ to 7.9 × 10^4^ M^−1^ cm^−1^, It displays that the combination of several heterocyclic conjugated aromatic units in dyes enhances the molar extinction coefficient. The light-harvesting ability of the synthesized compounds are confirmed to be higher due to the superior molar extinction coefficient values. The red shift in the absorption band and molar extinction coefficient resulted in improved efficiency of In-T-2C than In-B-C. The synthesized compound's emission spectra were documented in dimethyl sulfoxide (DMSO) at their excitation wavelength, which revealed high emission maxima observed between 447 nm and 530 nm because of the presence of the heterocyclic conjugated aromatic moieties. The stokes shift (SS, cm^−1^) and optical energy bandgap (*E*_g_, eV) were computed by applying eqn (1) and (2) (ref. 17) based on the UV-Visible and emission spectra. The *λ*_onset_ refers to the point at which the normalized UV-Visible and emission spectra intersect. The novel A-π-D-A structured dyes exhibit stokes shift values ranging between 3139–4497 cm^−1^. The In-T-2C dye exhibits a superior stokes shift value of 4497 cm^−1^, indicating an active electron transfer from the donor side to the acceptor moieties.^19^

1 SS = (10^7^/*λ*_Abs_) − (10^7^/*λ*_Em_)

2 *E*_g_ = 1240/*λ*_onset_

**Fig. 4:**
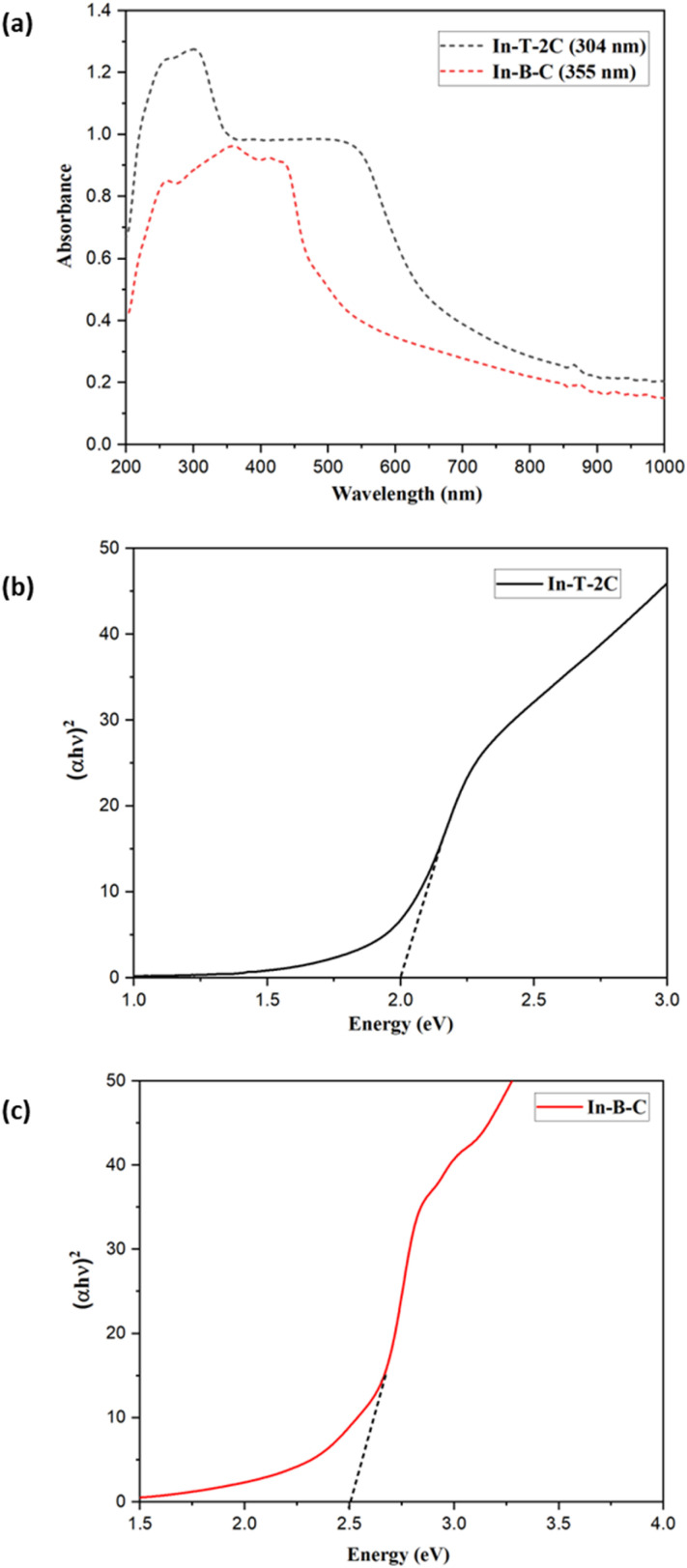
(a) Diffuse reflectance spectra and Tauc's plots of (b) In-T-2C and (c) In-B-C.

The diffuse reflectance spectra (DRS) were computed for the dyes In-T-2C and In-B-C. This spectrum enabled us to define the optical absorption or transmittance of the dyes. As demonstrated in Fig. 4a the charge transfer associated with In-T-2C and In-B-C was between 200 and 1000 nm, confirming that the absorption occurs in the UV-Visible region. The Kubelka–Munk eqn (3) was utilized to ascertain the absorption coefficient (*α*). The optical energy gap (*E*_g_) was determined by applying the eqn (4).^39,40^

3 (*α*/*S*) = (1 − *R*)^2^/2*R*

4 (*αhν*)^2^ = *A*(*hν* − *E*_g_)

where *α* is the absorption coefficient, *S* is the scattering coefficient, *R* is the diffused reflectance at specified energy, *h* is Plank's constant, *ν* is the frequency of incident photons and *A* is a constant. The optical energy gap (*E*_g_) of In-T-2C and In-B-C was examined using Tauc plot of (*αhν*)^2^*versus* photon energy (*hν*). The energy band gap (*E*_g_) obtained for In-T-2C and In-B-C compounds were 2.00 eV and 2.50 eV, because of the robust interactions of the molecules in the solid state, which restricts the effective charge transfer.^41^Fig. 4a and b demonstrates the diffuse reflectance spectra and Tauc plot of In-T-2C and In-B-C dyes.

### 2.3. Electrochemical properties

**Fig. 5:**
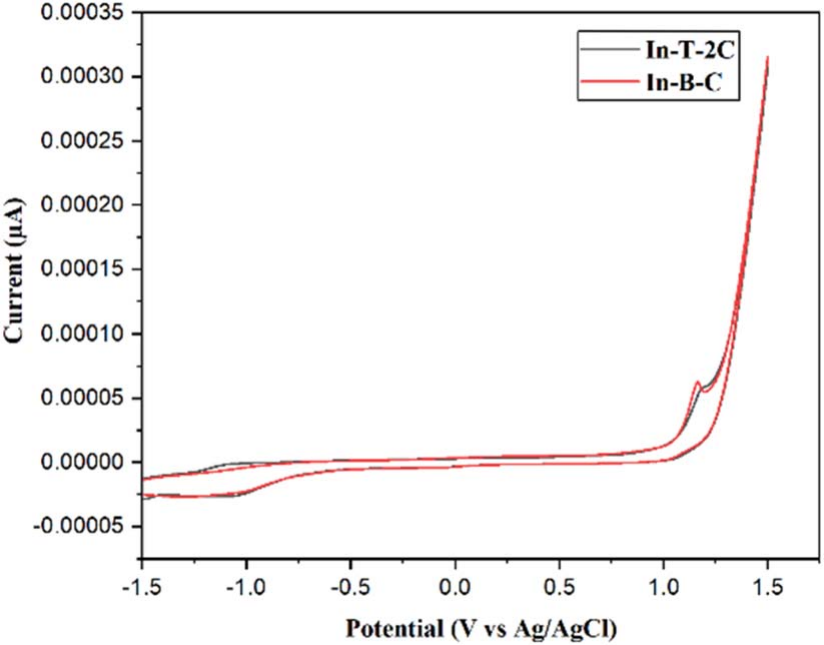
Cyclic voltammogram of A-π-D-A structured indole-based dyes.

The newly synthesized A-π-D-A structured indole-based dyes were subjected to cyclic voltammetric (CV) measurements to explore the achievability of electron injection to the conduction band (CB) of TiO_2_ from the dye's excited states.^42^ At a scan rate of 0.1 V s^−1^, the electrochemical traits of the synthesized molecules were assessed in dimethyl sulfoxide (DMSO) solution with 0.1 M tetrabutylammonium perchlorate (TBAP) serving as a supporting electrolyte. Ag/AgCl is used as the reference electrode, platinum as the counter electrode, and glassy carbon as the working electrode. Table 2 and Fig. 5 describe the details of electrochemical measurements and corresponding cyclic voltammograms of the synthesized moieties. In the present investigation, the potential of ferrocene/ferrocenium (Fc/Fc^+^) was determined to be 0.43 V to the Ag/AgCl electrode in the same circumstances. The energy level values of synthesized A-π-D-A structured indole-based dyes were computed by applying eqn (5) and (6).^41^

5 *E*_HOMO_ = −(*E*_ox_ + 4.4)

6 *E*_LUMO_ = (*E*_goptical_ + *E*_HOMO_)

**Table 2:** Electrochemical details of A-π-D-A structured indole-based metal-free dyes

In DMSO	*E*_ox_ (V)	*E*_HOMO_ (eV)	*E*_LUMO_ (eV)	*E*_g_ (eV)	Δ*G*_inj_	Δ*G*_reg_	Δ*G*_rec_
In-T-2C	1.177	−5.57	−2.98	2.59	−1.22	−0.37	−1.37
In-B-C	1.162	−5.56	−2.61	2.95	−1.59	−0.36	−1.36

The corresponding *E*_HOMO_ values in the respective oxidation states of In-T-2C and In-B-C were −5.57 and −5.56 eV. The redox potential of the I_3_^−^/I^−^ electrolyte is −5.2 eV, which is higher than these *E*_HOMO_ values. The *E*_LUMO_ of In-T-2C and In-B-C compounds appeared at higher energy levels of −2.98, and −2.61 eV in contrast to the conduction band energy level of TiO_2_ (−4.2 eV). It can be confirmed that the electrons are actively injected from the excited dye molecules into the conduction band of the TiO_2_ semiconductor.^10^ The energy band gap (*E*_g_) of In-T-2C and In-B-C compounds were 2.59 eV and 2.95 eV.

**Fig. 6:**
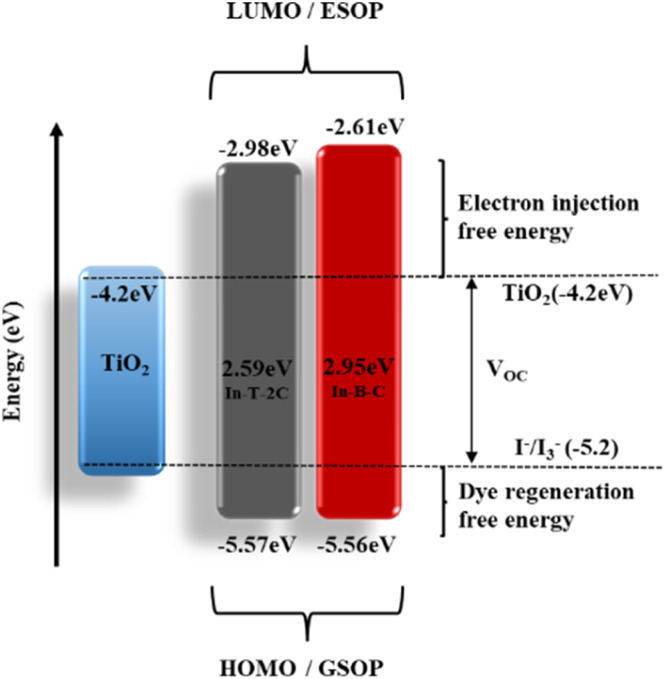
Energy level diagram for A-π-D-A structured indole-based dyes.

Furthermore, an energy level diagram derived from the synthesized A-π-D-A structured indole-based dye's HOMO and LUMO measurements is demonstrated in Fig. 6. Numerous electrochemical aspects, such as Gibbs free energies for electron injection (*G*_inj_), dye regeneration (*G*_reg_), and charge recombination (*G*_rec_) were calculated by applying eqn (7) and (8) to recognize the novel A-π-D-A structured compounds are appropriate to act as photosensitizers for dye-sensitized solar cells (DSSCs).^34^ In addition, the fact that the Gibbs free energies for the dye regeneration (*G*_reg_) as well as Gibbs free energies for electron injection (*G*_inj_) are negative in values, suggests the opportunity for electrolyte electron renewal and the thermodynamic electron injection into the TiO_2_ semiconductor's conduction band can be accomplished. On the other hand, the short circuit current (*J*_SC_) rises when the Gibbs free energy for electron injection (*G*_inj_) decreases.

7 Δ*G*_inj_ = LUMO − *E*_CB_ (TiO_2_)

8 Δ*G*_reg_ = *E* (I_3_^−^/I_2_^−^) − HOMO

9 Δ*G*_rec_ = *E*_CB_ (TiO_2_) − HOMO

### 2.4. Theoretical calculations

Using the B3LYP/6-31G (d, p) basis set in Gaussian 16 the density functional theory (DFT) and time-dependent density functional theory (TD-DFT) calculations were executed to probe the structural optimization, electron distribution, intramolecular charge transfer (ICT) effect and electronic absorption spectra of the synthesized A-π-D-A structured indole-based dyes.^21,33^

**Fig. 7:**
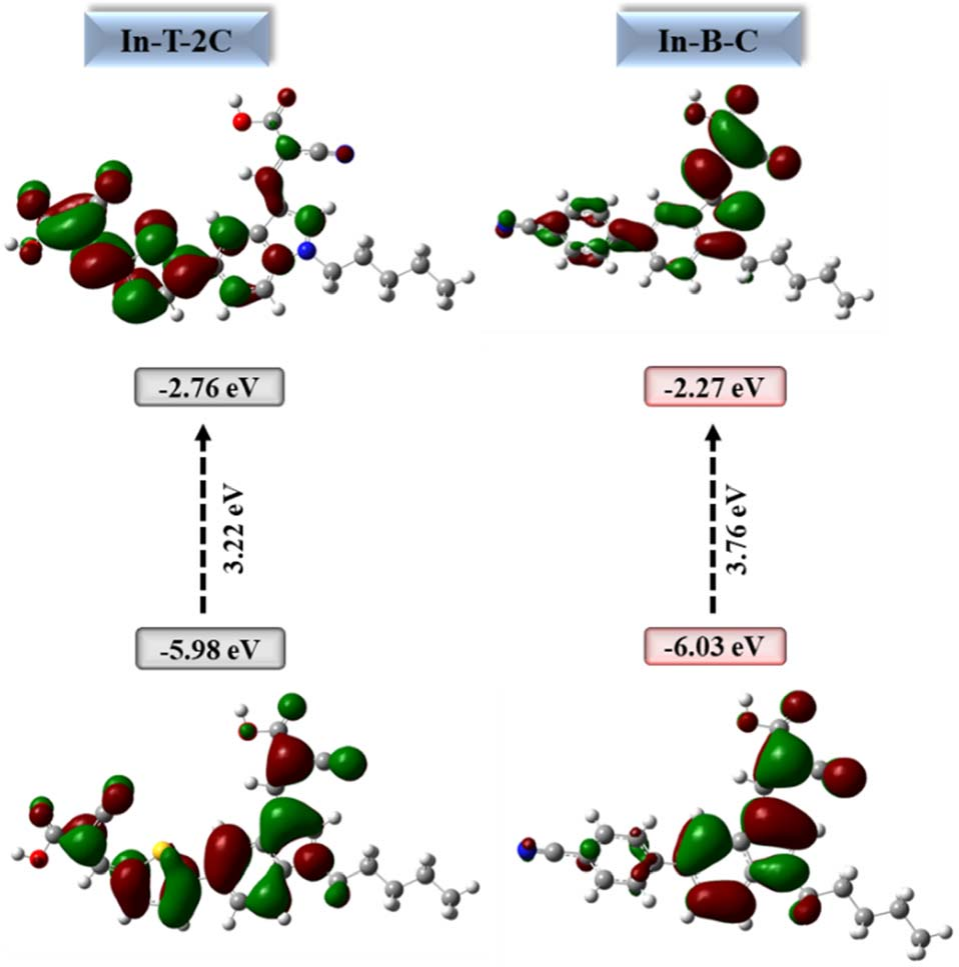
HOMOs, LUMOs and energy gap of A-π-D-A structured indole-based metal-free dyes.

Fig. 7 illustrates the distributions of electron density in the HOMO and LUMO energy levels of the new A-π-D-A structured indole-based dyes. In HOMO energy level of the In-T-2C, the electron density is primarily located on the donor indole group and the spacer thiophene unit. However, the electron cloud in the LUMO energy level of the In-T-2C compound is moved towards the linker thiophene moiety and acceptor cyanoacetic acid groups. It is noteworthy that an electron cloud flow appears to be favourably directed towards the acceptor cyanoacetic acid group which is linked to the indole unit through the π spacer thiophene moiety, because of the abundance of electrons in the π spacer.^43,44^ In the HOMO energy level of the In-B-C compound, the electron cloud is mainly concentrated on the donor indole group and in the LUMO energy level of the In-B-C compound is moved towards the spacer moiety and acceptor groups. Interestingly the electron cloud flow appears to be favourably directed towards the acceptor cyanoacetic acid group, because of the ability of the cyanoacetic acid group to accept electrons than the cyano group attached to the indole through the linker benzene unit. Computational studies results confirms that the corresponding HOMO energy levels of the A-π-D-A structured In-T-2C (−5.98 eV) and In-B-C (−6.03 eV) dyes are smaller than the I_3_^−^/I^−^ electrolyte redox potential (−5.2 eV), which encourages the process of dye regeneration. The LUMO of In-T-2C and In-B-C compounds seemed at higher energy levels of −2.76 and −2.27 eV, respectively, compared to the conduction band energy level of TiO_2_ (−4.2 eV). It can be confirmed that the electrons inject from the excited dye molecules into the TiO_2_ semiconductor's conduction band actively.^19^

The relevant parameters like ionization potential (IP), chemical potential (*μ*), electron affinity (EA), chemical hardness (*η*), electronegativity (*χ*), chemical softness (*σ*), electrophilicity index (*ω*), electron-accepting capacity (*ω*^+^) and nucleophilicity (*ε*) were used to explore the chemical reactivity as well as to identify the characteristics nature of the new A-π-D-A structured dyes as photosensitizers, by applying eqn (10)–(17).^26^ The ionization potential (IP) and electron affinity (EA) of the A-π-D-A structured dyes are closely linked to their HOMO and LUMO energy levels. The resistance of the compounds is measured by their chemical hardness, compounds that possess a higher number of conjugations exhibit lesser chemical hardness. Because of their lesser chemical hardness, they facilitate electron injection resulting in higher short-circuit current (*J*_SC_) values in DSSCs.^45^ The In-T-2C (1.61 eV) compound shows lower chemical hardness than the In-B-C (1.88 eV). Chemical potential is also known as the escaping tendency of the electron cloud, which demonstrates how easily electrons can be extracted from the donor moiety of the compounds and facilitates active charge transfer between the donor and acceptor units.^38^ The chemical potential values of novel In-T-2C and In-B-C dyes are 4.37 and 4.15 eV respectively. An additional method for figuring out the stabilization energy of the dyes is the electrophilicity index. This characteristic is also associated with the molecule's electron-accepting capacity. Higher electrophilicity index ratings indicate an easier charge transfer between the HOMO and LUMO levels of the compound.^26^ The In-T-2C (5.93 eV) shows a higher electrophilicity index than the In-B-C (4.58 eV). Based on the findings, it can be determined that In-T-2C is significantly superior to In-B-C dye. Table 3 describes the chemical parameters of newly synthesized A-π-D-A structured dyes.

10 Ionization potential = −*E*_HOMO_

11 Electron affinity = −*E*_LUMO_

12 Chemical potential (*μ*) = −electronegativity (*χ*) = (*E*_LUMO_ + *E*_HOMO_)/2

13 Chemical hardness (*η*) = (*E*_LUMO_ − *E*_HOMO_)/2

14 Chemical softness (*σ*) = 1/*η*

15 Electrophilicity index (*ω*) = *μ*^2^/2*η*

16 Nucleophilicity (*ε*) = 1/*ω*

17 Electron-accepting capacity (*ω*^+^) = (1 + 3 EA)^2^ /16(IP − EA)

**Table 3:** Chemical parameters of newly synthesized A-π-D-A structured indole-based dyes

Dyes	HOMO (eV)	LUMO (eV)	IP (eV)	EA (eV)	*χ* (eV)	*μ* (eV)	*η* (eV)	*σ* (eV)	*ω* (eV)	*ε* (eV)	*ω* ^+^ (eV)
In-T-2C	−5.98	−2.76	5.98	2.76	−4.37	4.37	1.61	0.62	5.93	0.16	1.67
In-B-C	−6.03	−2.27	6.03	2.27	−4.15	4.15	1.88	0.53	4.58	0.21	1.01

**Fig. 8:**
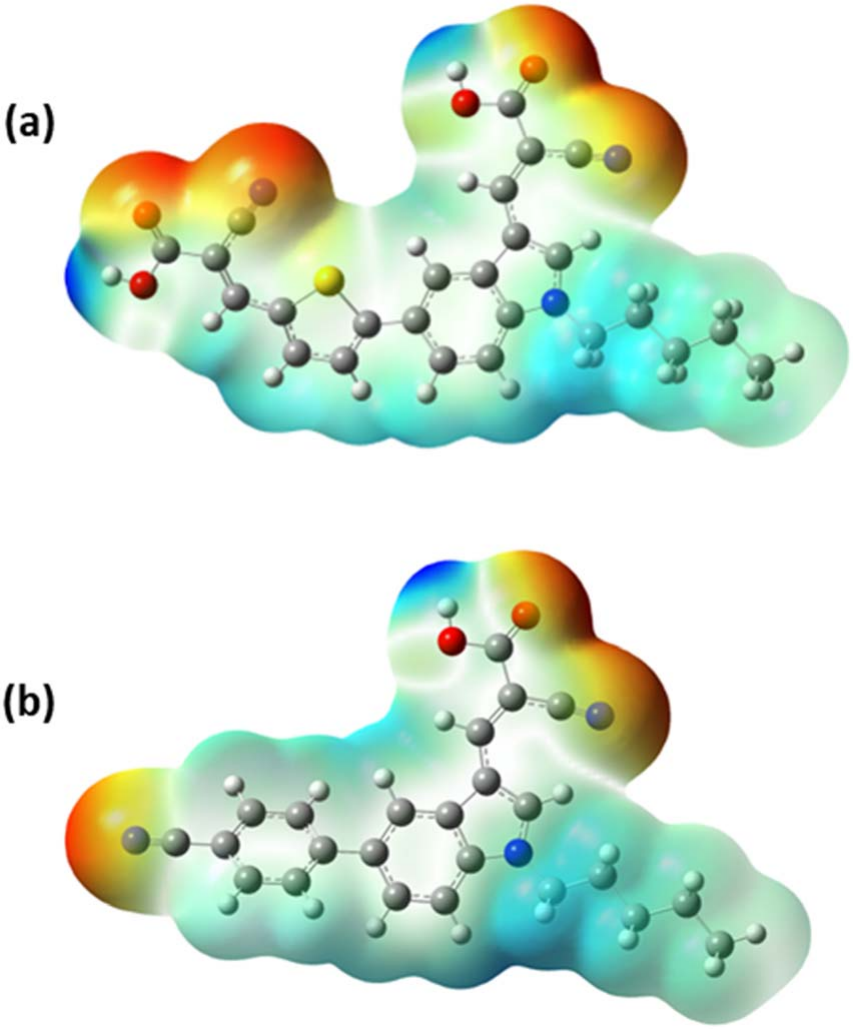
MESP surfaces of the A-π-D-A structured indole-based dyes (a) In-T-2C and (b) In-B-C.

The molecular electrostatic potential (MESP) analysis is an alternate technique for figuring out the electronic charge distribution and chemical reactivity of the developed A-π-D-A dyes in three-dimensional electron density surfaces. The mapped surfaces are expressed in a range of colours, from red to blue, corresponding to their electrostatic potential levels. An abundance of electrons and a deficiency of electrons are exhibited by red and blue regions representing low and high potentials respectively. The low potential region (red) and high potential region (blue) are also related to electrophilic and nucleophilic reactivity respectively.^46^ The red region is mostly localized on the electron-accepting moieties while the blue region lies on the donor and the spacer moieties of the developed A-π-D-A dyes.^38^ In both A-π-D-A structured dyes, the electron density is effectively delocalized from the donor unit to the acceptor moieties, encouraging the flow of electrons.^34^Fig. 8 demonstrates the MESP surfaces of the synthesized A-π-D-A structured dyes.

**Fig. 9:**
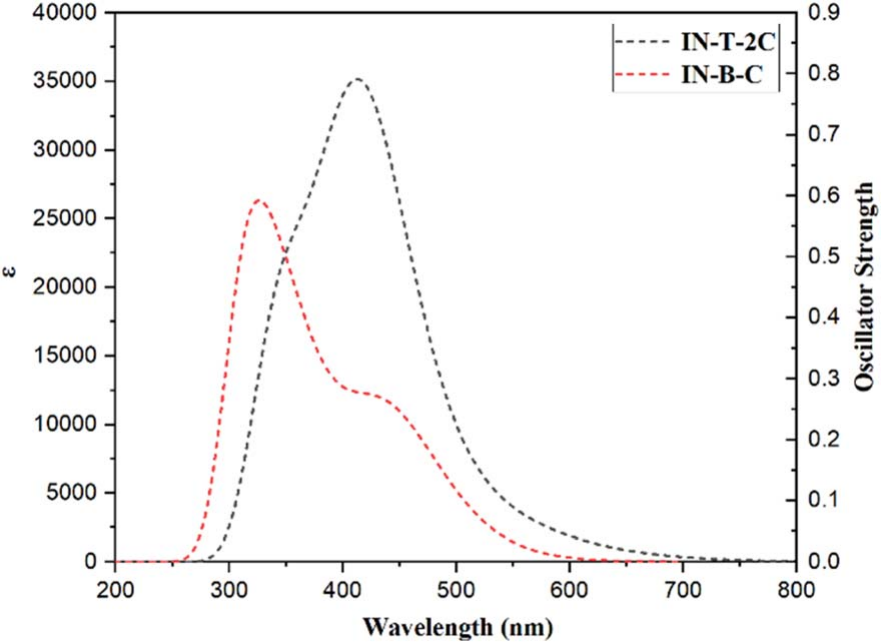
Theoretical absorption spectra of A-π-D-A structured indole-based dyes.

TD-DFT calculations were additionally used to evaluate the electronic transitions and UV-Visible spectra of the A-π-D-A structured dyes that were synthesized. The lowest energy transitions of the dyes were found to be correlated with HOMO to LUMO excitation, which was confirmed by the results of the TD-DFT calculations. The energy transfer percentages of HOMO to LUMO for In-T-2C and In-B-C compounds are 90% and 91%, respectively. The In-T-2C and In-B-C dyes have theoretical absorption maxima of 528 nm and 436 nm, respectively. The theoretical absorption maxima are mostly linked to the excitations resulting from the transitions from HOMO to LUMO. In comparison to the absorption peak detected in the experiment, the expected absorption bands of In-T-2C and In-B-C are a little overestimated. The literature has provided sufficient proof that the self-interaction error in TD-DFT studies can lead to an overestimation of the energies linked with the long-range charge transfer states.^19,47^Fig. 9 and Table 4 reveal the theoretical absorption spectra and TD-DFT calculation details. The light-harvesting efficiency (LHE) of the synthesized A-π-D-A structured dyes In-T-2C and In-B-C are 0.142 and 0.451, respectively.^48^ Moreover, to estimate the open circuit voltage (*V*_OC_), the difference between the LUMO energy level and the conduction band (CB) of the TiO_2_ semiconductor was utilized. The light-harvesting efficiency (LHE) and open circuit voltage (*V*_OC_) were figured out using eqn (18) and (19).

18 LHE = 1–10^−*f*^

19 *V*_oc_ = *E*_LUMO_ − *E*_CB_

**Table 4:** TD-DFT data for A-π-D-A structured indole-based dyes

Dyes	*λ*_max_ (nm)	Composition (%)	*E* (eV)	*F*	LHE	*V*_OC_
In-T-2C	528	H-1 → L (9%), H → L (90%)	2.86	0.067	0.142	1.44
	418	H-1 → L (79%), H → L (7%), H → L + 1 (11%)	3.14	0.810	0.845	
	347	H-1 → L (6%), H-1 → L + 1 (26%), H → L + 1 (63%)	3.50	0.458	0.373	
In-B-C	439	H-1 → L (6%), H → L (91%)	3.48	0.261	0.451	1.93
	359	H-1 → L (83%), H → L (3%), H → L + 1 (9%)	3.75	0.310	0.510	
	317	H-1 → L (5%), H-1 → L + 1 (3%), H → L (3%), H → L + 1 (83%)	3.96	0.538	0.710	

### 2.5. Photovoltaic performance of DSSCs

The photovoltaic study of the fabricated dye-sensitized solar cells (DSSCs) sensitized by the N719 dye and novel A-π-D-A structured dyes was investigated under the illumination of 1 Sun solar simulator (100 mW cm^−2^). The FTO glass was utilized to fabricate DSSCs, a layer of TiO_2_ was coated on it and used as a photoanode (working electrode). The photoanode was submerged in a solution containing the synthesized dyes (3 × 10^−4^ M), which enabled the dyes to be anchored on the surface of TiO_2_ under dark conditions. For the preparation of the counter electrode, a platinum solution of 2 mM Chloroplatinic acid hexahydrate in isopropyl alcohol was drop cast on the FTO glass and annealed at 450 °C. After sealing the two electrodes, the device was injected with an I^−^/I_3_^−^ electrolyte solution.^30^

**Fig. 10:**
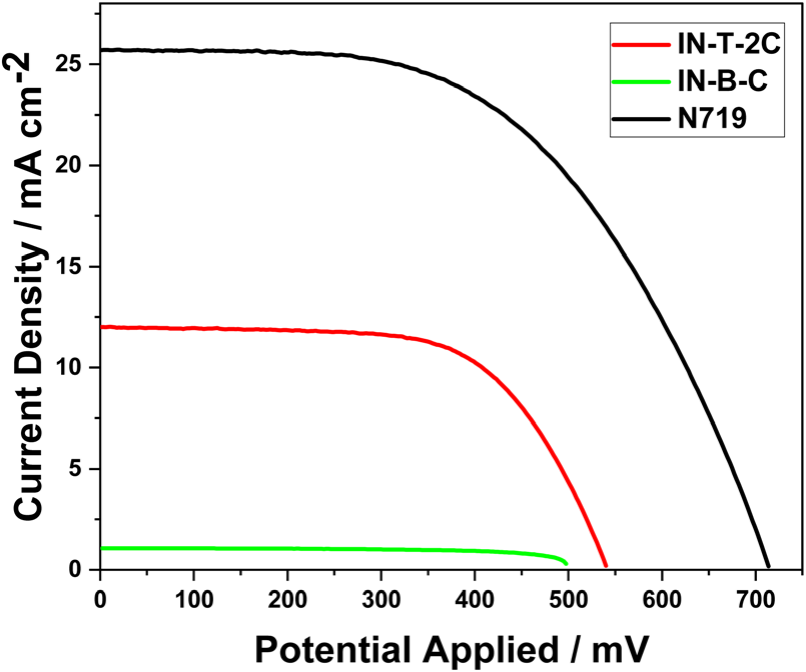
The current density–voltage characteristics of DSSCs using In-T-2C, In-B-C and N719 dye.

The device sensitized using In-T-2C dye demonstrated a better photovoltaic performance than the device with In-B-C dye. In-T-2C dye-based DSSC device exhibited the fill factor (FF) of 0.63, resulting in the highest open-circuit voltage (*V*_OC_) of 540.2 mV and highest efficiency (*η*) of 4.12% due to the highest short-circuit current density (*J*_SC_) of 12.1 mA cm^−2^, compared to In-B-C dye (*V*_OC_ = 497 mV, *J*_SC_ = 1.07 mA cm^−2^, FF = 0.70, *η* = 0.38%). The N719-sensitized DSSC was also constructed and tested in comparable conditions. The device, based on the N719, displays the open-circuit voltage (*V*_OC_) of 713.8 mV, short-circuit current density (*J*_SC_) of 25.7 mA cm^−2^, fill factor (FF) of 0.54 and power conversion efficiency (*η*) of 9.91%. Unfortunately, the In-B-C dye-sensitized system did not show a better photovoltaic performance. Benzene is the spacer (π) unit present in the In-B-C dye and thiophene moiety in the In-T-2C dye, which affects the electron injection process as well as the power conversion efficiency of the solar cells.^25,49^ Moreover, the phenyl group connected to the indole moiety can rotate, which leads to the breakage of conjugation and reduces the intramolecular charge transfer (ICT) between the donor and the acceptor moieties.^50^Fig. 10 displays the current density–voltage curve of dye-sensitized solar cells (DSSCs) that have been sensitized using In-T-2C, In-B-C, and N719 dye. Table 5 provides information on the photovoltaic parameters of the cell, such as short-circuit current (*J*_SC_), open-circuit voltage (*V*_OC_), fill factor (FF), and photocurrent efficiency (*η*). The following equations were used to calculate the fill factor (FF) (eqn (20)) and power conversion efficiency (PCE) (eqn (21)).^51^

20 FF = (*V*_max_ × *J*_max_)/(*V*_OC_ × *J*_SC_)

21 *η* = (*J*_SC_ × *V*_OC_ × FF)/*P*_in_

where *V*_max_ is the maximum voltage, and *J*_max_ is the maximum current at peak power intensity. ^46^ The power conversion efficiency (PCE) of a solar cell is the percentage ratio of electrical power produced to the optical power influencing the cell.^52^

**Table 5:** Photovoltaic parameters of DSSCs using In-T-2C. In-B-C and N719 dye

Dyes	*V*_OC_ (mV)	*J*_SC_ (mA cm^−2^)	FF	*η* (%)	*R*_s_ (Ω)	*R*_CT_ (Ω)	Electron lifetime (*τ*) (ms)
In-T-2C	540.2	12.1	0.63	4.12	4.44	6.24	4.74
In-B-C	497.6	1.07	0.70	0.38	3.93	143.60	20.51
N719	713.8	25.7	0.54	9.91	4.87	6.96	4.72

### 2.6. Electrochemical impedance analysis

**Fig. 11:**
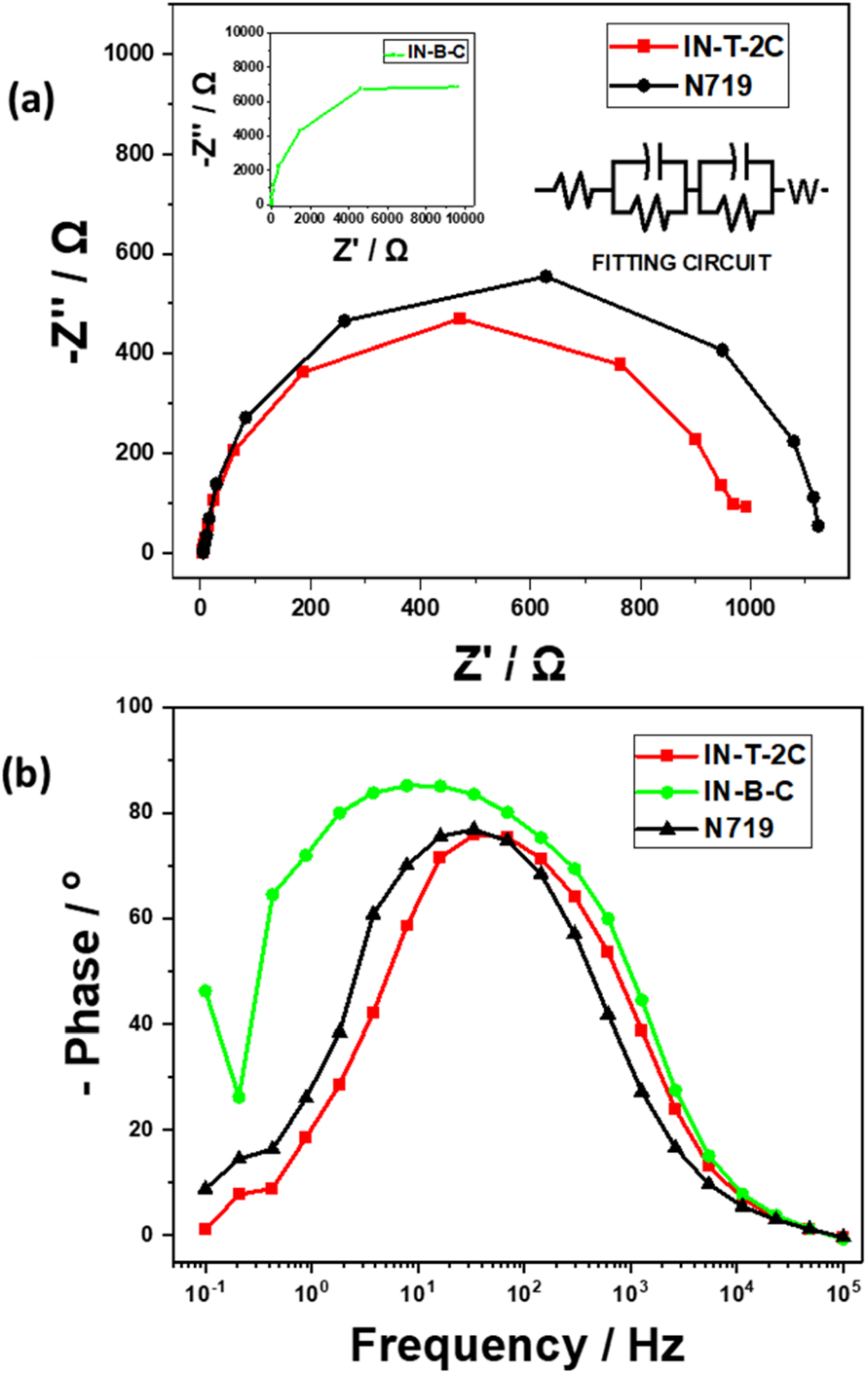
(a) Nyquist plots and (b) Bode plots of DSSCs using In-T-2C, In-B-C and N719 dye.

An electrochemical impedance spectroscopy (EIS) investigation was conducted to determine the synthesized dye's electron lifetime, the causes of the high *V*_OC_ values, and the process of interfacial electron transfer within DSSCs. Fig. 11a displays the Nyquist plots of the dye-sensitized solar cells (DSSCs) that have been sensitized using In-T-2C, In-B-C, and N719 dye. The analyzed bigger semicircle in the Nyquist plot reflects the charge transfer resistance at the TiO_2_/dye/electrolyte interface.^53^ A notable difference in the radius of the semicircles related to charge transfer resistance can be seen in the Nyquist plot when the TiO_2_ surface gets coated by various dyes. The semicircle associated with charge transfer at the TiO_2_/dye/electrolyte interface is larger; its charge transfer resistance will be greater, as well as provide better *V*_OC_ values.^54^Fig. 11a demonstrates that the charge transfer resistance (*R*_CT_) increased in the following order: In-T-2C (6.24 Ω) < N719 (6.96 Ω), which appears by the trend of *V*_OC_ given in Table 5.

The electron lifetime (*τ*) of the dye-sensitized solar cells (DSSCs) with In-T-2C, In-B-C, and N719 dyes were examined using Bode plots. It can be measured using the following eqn (22),

22 Electron lifetime (*τ*) = 1/(2π*f*)

The electron lifetime (*τ*) of the In-T-2C dye in the conduction band of TiO_2_ is 4.74 ms, and for the N719 dye is 4.72 ms. In general, an extended electron lifetime in the TiO_2_ conduction band is advantageous since it diminishes the back-reaction between the injected electrons and the electrolyte.^19^ The material In-B-C showed an acceptable *V*_OC_ (497.6 mV) and better FF (0.70), *R*_S_ (3.93 Ω), and *τ* (20.51 ms) values but poor PCE (0.38%) because of the lower *J*_SC_ (1.07 mA cm^−2^) obtained for the cell fabricated with In-B-C as dye-sensitizer, which is also established by the much higher *R*_CT_ (143.60 Ω) value. This is because of the spacer (π) unit (Benzene) present in the In-B-C dye, which makes the dye less sensitizing and makes the overall cell less conductive in nature.

So, by incorporating all the properties of dyes, photophysical properties demonstrated that the molar extinction coefficient values of these dyes are astonishingly high at peaks responsible for the intramolecular charge transfer (ICT). The UV-DRS spectra of the compounds on the TiO_2_ surface disclose that the absorption maxima for In-T-2C dye were red-shifted and In-B-C dye was blue-shifted compared to their absorption maxima in DMSO. The molar extinction coefficient values and UV-DRS spectra of the dyes reveal the higher ability of the dyes to act as photosensitizers in DSSCs. Various factors can influence the performance of a dye in DSSC despite its good photophysical properties. The efficiency of electron injection from the photosensitizer to TiO_2_ is critical. The *E*_LUMO_ of In-T-2C and In-B-C compounds appeared at higher energy levels of −2.98, and −2.61 eV in contrast to the conduction band energy level of TiO_2_ (−4.2 eV), which affects the electron injection efficiency.^9,55^ As well as dye's narrow absorption in the range of visible regions reduces the absorption of sunlight, which also affects the performance of DSSC.^34^ In computational investigation, the LUMO energy level of the In-B-C, the electron cloud flow appears to be favorably directed towards the acceptor cyanoacetic acid group rather than the cyano group attached to the indole through the linker benzene unit. This effect might also affect the performance. When compared with the reported simple metal-free organic dyes, like 1,6-bis[3-((*E*)-2-carboxyl-2-cyanovinyl)indol-1-yl] hexane ((D-A)_2_)^55^ demonstrated efficiency (*η*) of 1.19%, 2-cyano-3-(1-(10-hexyl-10*H*-phenothiazin-3-yl)-1*H*-indol-3-yl)acrylic acid (D-π-A)^13^ proved efficiency (*η*) of 3.30%, 4-((*E*)-(5-((Z)-1-cyano-2-(1-hexyl-1*H*-indol-3-yl)vinyl)thiophen-2-yl)methyleneamino)benzoic acid (D-π-A)^15^ reported efficiency (*η*) of 1.18%, 3-(6-(-2-carboxy-2-cyanovinyl)-9-hexyl-9*H*-carbazol-3-yl)-2-cyanoacrylic acid (D-π-A)^9^ showed efficiency (*η*) of 1.06% and 6-(2-cyano-2-(5-(2,2-dicyanovinyl)thiophen-2-yl)vinyl)-9-(2-ethylhexyl)-9*H*-carbazole-3-carboxylic acid (D-A-π-A′)^31^ demonstrated efficiency (*η*) of 0.027%. However, the novel In-T-2C (A-π-D-A) dye reported here showed comparatively better efficiency because of the presence of thiophene moiety as a spacer and two cyanoacetic acid moieties as acceptor group.

## 3 Experimental

### 3.1. Materials and methods

The solvents and chemicals employed for the present investigation were all purchased from TCI, Spectrochem, Avra, and Sigma Aldrich; they were all used exactly as received, without undergoing any purification. Using a BRUKER (400 MHz) spectrometer, the ^1^H and ^13^C spectra were recorded in δ (ppm) with tetramethyl silane serving as an internal standard and DMSO-*d*_6_ and CDCl_3_ as solvent. Employing the ESI method on the WATERS-XEVO G2-XS-QToF High-Resolution Mass Spectrometer (HRMS), the HRMS spectral data were recorded. PerkinElmer RX-IFT-IR spectrometer was used to capture the FT-IR spectra, and the absorbance readings are in cm^−1^. For the optical studies, the JASCO UV-visible spectrometer and HITACHI Fluorescence spectrometer were utilized. Cyclic voltammograms of the prepared samples were documented using a CH-Instruments Model CHI620E. The electrochemical analysis of *J*–*V* characteristics and electrochemical impedance characteristics were performed using the Metrohm AUTOLAB12/FRA2 PGSTAT302N electrochemical analyzer, associated with the 1 Sun solar simulator (100 mW cm^−2^), with the fabricated DSSC.

### 3.2. Synthesis

The synthetic procedure implemented for developing the intended A-π-D-A kind structured dyes by utilizing indole is described in Scheme 1.

#### 3.2.1. Synthesis of 5-bromo-1-pentyl-1*H*-indole-3-carbaldehyde (1a) compound

1-bromopentane (3.69 g, 0.0222 mol), potassium carbonate (3.68 g, 0.0266 mol) and 5-bromoindole-3-carboxaldehyde (2 g, 0.0088 mol) were combined in 4 ml of dimethylformamide (DMF) and left to stir for 2 hours in room temperature. The reaction mixture was then extracted by using ethyl acetate and double-distilled water after the reaction mixture had been neutralized with dil. HCl (10 N, 2 ml). By utilizing the anhydrous magnesium sulfate (MgSO_4_) the organic phase was dried out. The excess solvent evaporated and cooled to room temperature.^15,33,55^

##### 3.2.1.1 5-Bromo-1-pentyl-1*H*-indole-3-carbaldehyde (1a)

Cream colour solid. Yield-95%. Melting point-75 °C–79 °C. IR, *ν*, cm^−1^: 3109, 3041, 2950, 2864, 2822, 1851, 1779, 1725, 1648, 1606, 1566, 1528, 1455, 1409, 1349, 1292, 1230, 1198, 1138, 1112, 1047, 1025, 876, 825, 791, 745, 642, 617, 591, 511. ^1^H NMR (400 MHz, δ, ppm) (CDCl_3_): 9.94 (s, 1H), 8.45 (s, 1H), 7.69 (s, 1H), 7.40 (d, *J* = 8.6 Hz, 1H), 7.22 (d, *J* = 8.6 Hz, 1H), 4.13 (t, *J* = 7.1 Hz, 2H), 1.87 (t, *J* = 7.2 Hz, 2H), 1.33 (m, 4H), 0.89 (t, *J* = 6.8 Hz, 3H). ^13^C NMR (100 MHz, δ, ppm): 184.23, 138.67, 135.87, 126.91, 124.78, 117.39, 116.50, 111.52, 77.36, 77.25, 77.05, 76.73, 47.49, 30.94, 29.43, 28.91, 22.21, 13.88.

#### 3.2.2. Synthesis of 5-(5-formylthiophen-2-yl)-1-pentyl-1*H*-indole-3-carbaldehyde (2b) and 4-(3-formyl-1-pentyl-1*H*-indol-5- yl)benzonitrile (2c) compounds

5-Formyl-2-thienylboronic acid (1.59 g, 0.0101 mol), potassium carbonate (1.87 g, 0.0135 mol), 5-bromo-1-pentyl-1*H*-indole-3-carbaldehyde (2 g, 0.0067 mol) and [1,1′-bis(diphenylphosphino)ferrocene]dichloropalladium(ii) [Pd(dppf)Cl_2_] (5 mol%) were all dissolved in a mixture of 1,4 dioxane and water in a 3 : 1 ratio. The reaction mixture was placed in a Schlenk flask and heated at 70 °C for 22 hours while exposed to nitrogen. After allowing the reaction mixture to reach room temperature, it was filtered through a Celite bed and the product 2b was extracted using ethyl acetate and double-distilled water. The organic phase was dried by utilizing anhydrous magnesium sulfate (MgSO_4_), and the excess solvent was removed by evaporating it.^56,57^ Similarly, we synthesized 4-(3-formyl-1-pentyl-1*H*-indol-5-yl)benzonitrile (2c) using 4-cyanophenylboronic acid.

##### 3.2.2.1 5-(5-Formylthiophen-2-yl)-1-pentyl-1*H*-indole-3-carbaldehyde (2b)

Brown colour solid. Yield-71%. Melting point-110 °C–112 °C. IR, *ν*, cm^−1^: 3292, 3107, 2944, 2850, 1651, 1528, 1437, 1386, 1230, 1173, 1150, 1099, 1059, 1036, 988, 928, 883, 788, 751, 717, 665, 646, 614, 589. ^1^H NMR (400 MHz, δ, ppm) (DMSO-*d*_6_): 9.95 (s, 1H), 9.90 (s, 1H), 8.48 (s, 1H), 8.42 (s, 1H), 8.03 (d, *J* = 3.9 Hz, 1H), 7.76 (s, 2H), 7.72 (d, *J* = 3.8 Hz, 1H), 4.30 (t, *J* = 7 Hz, 2H), 1.82 (t, *J* = 7.1 Hz, 2H), 1.30 (m, 4H), 0.84 (t, *J* = 3.5 Hz, 3H). ^13^C NMR (100 MHz, δ, ppm): 185.25, 184.33, 154.32, 142.26, 141.82, 139.91, 138.02, 127.58, 125.11, 122.62, 119.15, 117.73, 112.65, 40.94, 31.14, 28.69, 22.14, 14.28.

##### 3.2.2.2 4-(3-Formyl-1-pentyl-1*H*-indol-5-yl)benzonitrile (2c)

Light brown solid. Yield-73%. Melting point-140 °C–142 °C. IR, *ν*, cm^−1^: 3126, 2930, 2862, 2218, 1657, 1606, 1526, 1463, 1397, 1352, 1301, 1232, 1181, 1112, 1019, 902, 856, 816, 785, 751, 702, 697, 654, 600, 557, 523. ^1^H NMR (400 MHz, δ, ppm) (CDCl_3_): 10.02 (s, 1H), 8.56 (s, 1H), 7.77 (d, *J* = 9.4 Hz, 3H), 7.70 (d, *J* = 8.3 Hz, 2H), 7.56 (d, *J* = 8.5 Hz, 1H), 7.47 (d, *J* = 8.5 Hz, 1H), 4.21 (t, *J* = 7 Hz, 2H), 1.92 (t, *J* = 6.7 Hz, 2H), 1.36 (d, *J* = 3 Hz, 4H), 0.91 (t, *J* = 6.5 Hz, 3H). ^13^C NMR (100 MHz, δ, ppm): 184.56, 146.12, 139.34, 137.33, 134.22, 132.52, 127.99, 126.04, 123.37, 121.14, 119.15, 118.36, 110.78, 110.32, 77.39, 77.27, 77.07, 76.75, 47.51, 30.94, 29.53, 28.94, 22.24, 13.90.

#### 3.2.3. Synthesis of 3-(5-(3-((2-carboxy-2-cyanovinyl)-1-pentyl-1*H*-indol-5-yl)thiophen-2-yl)-2-isocyanoacrylic acid) (In-T-2C) and 2-cyano-3-(5-(4-cyanophenyl)-1-pentyl-1*H*-indol-3-yl)acrylic acid (In-B-C)

Compound 5-(5-formylthiophen-2-yl)-1-pentyl-1*H*-indole-3-carbaldehyde (2b) (2 g, 0.0061 mol), piperidine (0.9 ml) and cyanoacetic acid (1.56 g, 0.0184 mol), were combined in ethanol (6 ml) and was refluxed for 5 hours at 79 °C. Ethyl acetate and double-distilled water were used to extract the product In-T-2C after neutralizing the reaction mixture with dilute HCl (10 N, 2 ml). The excess solvent was evaporated after drying the organic phase with anhydrous magnesium sulfate (MgSO_4_).^25,58^ Likewise, we synthesized 2-cyano-3-(5-(4-cyanophenyl)-1-pentyl-1*H*-indol-3-yl)acrylic acid (In-B-C) compound using 4-(3-formyl-1-pentyl-1*H*-indol-5-yl)benzonitrile (2c).

##### 3.2.3.1 3-(5-(3-((2-Carboxy-2-cyanovinyl)-1-pentyl-1*H*-indol-5-yl)thiophen-2-yl)-2-isocyanoacrylic acid) (In-T-2C)

Red colour solid. Yield-73%. Melting point-215 °C–220 °C. IR, *ν*, cm^−1^: 3111, 2930, 2864, 2577, 2215, 1697, 1571, 1515, 1457, 1426, 1396, 1349, 1306, 1210, 1181, 1130, 1070, 1025, 945, 859, 800, 760, 720, 662, 591, 563. ^1^H NMR (400 MHz, δ, ppm) (DMSO-*d*_6_): 8.64 (s, 1H), 8.58 (s, 1H), 8.48 (s, 1H), 8.02 (d, *J* = 4 Hz, 1H), 7.88 (d, *J* = 4 Hz, 1H), 7.78 (d, *J* = 8 Hz, 1H), 7.72 (d, *J* = 8 Hz, 1H), 4.38 (t, *J* = 6 Hz, 2H), 1.83 (m, 2H), 1.30 (m, 4H), 0.85 (t, *J* = 8 Hz, 3H). ^13^C NMR (100 MHz, δ, ppm): 164.98, 164.20, 154.64, 147.17, 145.95, 142.03, 137.18, 134.55, 128.62, 127.23, 125.45, 122.62, 118.71, 117.67, 117.06, 112.97, 110.15, 98.14, 95.29, 47.27, 29.62, 28.67, 22.11, 14.29. HRMS (ESI) *m*/*z*: [M + H]^+^ calculated for C_25_H_21_N_3_O_4_S is 460.1331, found 460.0851.

##### 3.2.3.2 2-Cyano-3-(5-(4-cyanophenyl)-1-pentyl-1*H*-indol-3-yl)acrylic acid (In-B-C)

Greenish yellow solid. Yield-81%. Melting point-223 °C–230 °C. IR, *ν*, cm^−1^: 3109, 2941, 2867, 2221, 1688, 1571, 1520, 1471, 1400, 1343, 1272, 1184, 1127, 1013, 942, 873, 839, 800, 765, 722, 685, 651, 606, 549. ^1^H NMR (400 MHz, δ, ppm) (DMSO-*d*_6_): 8.64 (s, 1H), 8.58 (s, 1H), 8.48 (s, 1H), 8.02 (d, *J* = 4 Hz, 1H), 7.89 (d, *J* = 4 Hz, 1H), 7.78 (d, *J* = 8 Hz, 1H), 7.72 (d, *J* = 8 Hz, 1H), 4.38 (t, *J* = 6 Hz, 2H), 1.83 (m, 2H), 1.30 (m, 4H), 0.85 (t, *J* = 8 Hz, 3H). ^13^C NMR (100 MHz, δ, ppm): 165.18, 146.19, 141.13, 136.21, 135.39, 135.22, 129.32, 128.57, 127.49, 123.27, 118.96, 117.63, 112.35, 109.95, 94.43, 47.23, 31.15, 31.13, 29.82, 26.15, 22.45, 14.29. HRMS (ESI) *m*/*z*: [M + H]^+^ calculated for C_24_H_21_N_3_O_2_ is 384.1712, found 384.1716.

### 3.3. Fabrication of DSSCs

The purchased FTO plates were cut into 2 × 2 cm^2^, immersed in soap solution for 10 minutes, and sonicated. Subsequently, the FTO plates were sonicated in solvents and cleaned with deionized water, acetone, and isopropyl alcohol. After that, they were soaked in a 40 mM TiCl_4_ bath at 80 °C for 40 minutes and allowed to cool to room temperature. After treating the FTO plates with TiCl_4_, they were washed with water and ethanol and dried in a hot air oven for 30 minutes at 80 °C.

In an amber culture tube, about 0.2 g of TiO_2_ powder was added, along with 15 mg of PEG, 0.5 ml of acetylacetone, three drops of Triton-X, and 0.5 ml of deionized water. Aluminium foil is used to wrap the vial, followed by an hour of sonication. After that, stir gently for 24 hours with a magnetic stirrer. The prepared TiO_2_ paste was coated on the FTO plates using the doctor-blade method *via* a 55 μm thick scotch tape. Subsequently, it was annealed at 450 °C in an annealing oven and allowed to cool to room temperature. In a 100 ml standard measuring flask, 3 × 10^− 4^ M of dyes were dissolved in a mixture of acetonitrile and tertiary butanol with a ratio of 1 : 1, and the mixture was sonicated for 30 minutes. The TiO_2_-coated FTO plates were immersed in the dye solution for 24 h in dark conditions. Then the coated plates were washed with water and ethanol to remove the excess dye. For the preparation of the counter electrode, a platinum solution of 2 mM chloroplatinic acid hexahydrate in isopropyl alcohol was drop cast on the FTO glass and annealed at 450 °C. The dye-adsorbed photoanode and counter electrode are sandwiched between each other and held together by binder clips. Parafilm is used to cover the excess area to prevent short circuits between the electrodes. Using a micropipette, 20 μL of the I^−^/I_3_^−^ electrolyte is injected between the electrodes. The electrolyte is prepared by using 0.05 M of Iodine, 0.5 M of lithium Iodide, and 0.5 M of 4-tertiary butyl pyridine in a 10 ml standard measuring flask with 3-methoxypropionitrile.^51^

## 4 Conclusions

In conclusion, unique A-π-D-A structured metal-free indole-based dyes were designed and developed. The novel 3-(5-(3-((2-carboxy-2-cyanovinyl)-1-pentyl-1*H*-indol-5-yl)thiophen-2-yl)-2-isocyanoacrylic acid) (In-T-2C) and 2-cyano-3-(5-(4-cyanophenyl)-1-pentyl-1*H*-indol-3-yl)acrylic acid (In-B-C), depicted two significant absorption bands in the range of 281–377 nm and 392–428 nm. It discovered that the absorption band responsible for the intramolecular charge transfer (ICT) in the In-T-2C compound is red-shifted than the In-B-C compound. The In-T-2C dye exhibits a maximum stokes shift value of 4497 cm^−1^, indicating an active electron transfer from the donor to the acceptor moieties. In-T-2C on TiO_2_ film demonstrates a red shift and a broader absorption band, which indicates it has a robust light harvesting ability on the TiO_2_ surface. The energy band gap (*E*_g_) of In-T-2C and In-B-C were 2.59 eV and 2.95 eV. Density functional theory (DFT) and time-dependent density functional theory (TD-DFT) were executed to acquire an in-depth comprehension of the geometry, electronic structure and absorption spectra of the newly synthesized dyes. The energy transfer percentages of HOMO to LUMO in In-T-2C and In-B-C compounds are 90% and 91%, respectively. The system sensitized using In-T-2C dye demonstrated a better photovoltaic performance than the device with In-B-C dye. In-T-2C dye-based DSSC device exhibited the fill factor (FF) of 0.63, resulting in the highest open-circuit voltage (*V*_OC_) of 540.2 mV and highest efficiency (*η*) of 4.12% due to the highest short-circuit current density (*J*_SC_) of 12.1 mA cm^−2^, compared to In-B-C dye (*V*_OC_ = 497 mV, *J*_SC_ = 1.07, FF = 0.70, *η* = 0.38%). Electrochemical impedance spectroscopy (EIS) was performed to recognize the interfacial charge transfer as well as recombination in the dye-sensitized solar cells (DSSCs). The electron lifetime (*τ*) of the In-T-2C dye in the conduction band of TiO_2_ is 4.74 ms, and for the N719 dye is 4.72 ms. The findings establish that the indole-based A-π-D-A type In-T-2C dye is suitable for dye-sensitized solar cells (DSSCs) with better photovoltaic activity.

## Data availability

The data supporting this article have been included as part of the ESI.†

## Author contributions

Krupa Elsa Roys: method development, experimental section, investigation, writing – original draft preparation. Manju S. L: conceptualization, methodology, supervision, validation. Mohamed Siddiq: experimental section, writing – original draft preparation. Anandan Sambandam: conceptualization, supervision.

## Conflicts of interest

The authors declare that they have no financial or personal relationships that are known to them that would appear to have influenced the scientific work described in this study.

## Supplementary Material

RA-014-D4RA05341A-s001

RA-014-D4RA05341A-s002
