# Supplementary material for: Novel A-π-D-A organic dyes for better photovoltaic performance
Source: RSC Adv. 2024 Sep 16;14(40):29229–41. doi: 10.1039/d4ra05341a (PMC11403392; doi:10.1039/d4ra05341a)
Supplement: RA-014-D4RA05341A-s001 [file RA-014-D4RA05341A-s001.pdf]

Supplementary Information (SI) for RSC Advances.
This journal is © The Royal Society of Chemistry 2024

Novel A-π-D-A organic dyes for better Photovoltaic performance

Krupa Elsa Roys[a], Manju S L[a,*], Mohamed Siddiq[b], Anandan Sambandam[b].

[a]*Department of Chemistry, School of Advanced Sciences, Vellore Institute of Technology, Vellore-632014, India. Email: slmanju@vit.ac.in*

[b]*Nanomaterials and Solar Energy Conversion Laboratory, Department of Chemistry, National Institute of Technology, Trichy- 620015, India. Email: sanand@nitt.edu*

Scanned copies of spectra (FTIR, ^{1}H and ^{13}C NMR, HRMS)

1. 3-(5-(3-((2-carboxy-2-cyanovinyl)-1-pentyl-1*H*-indol-5-yl)thiophen-2-yl)-2-isocyanoacrylic acid (In-T-2C)

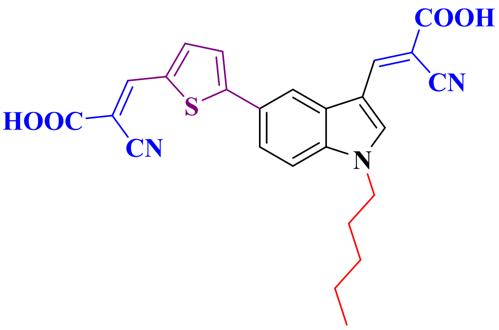

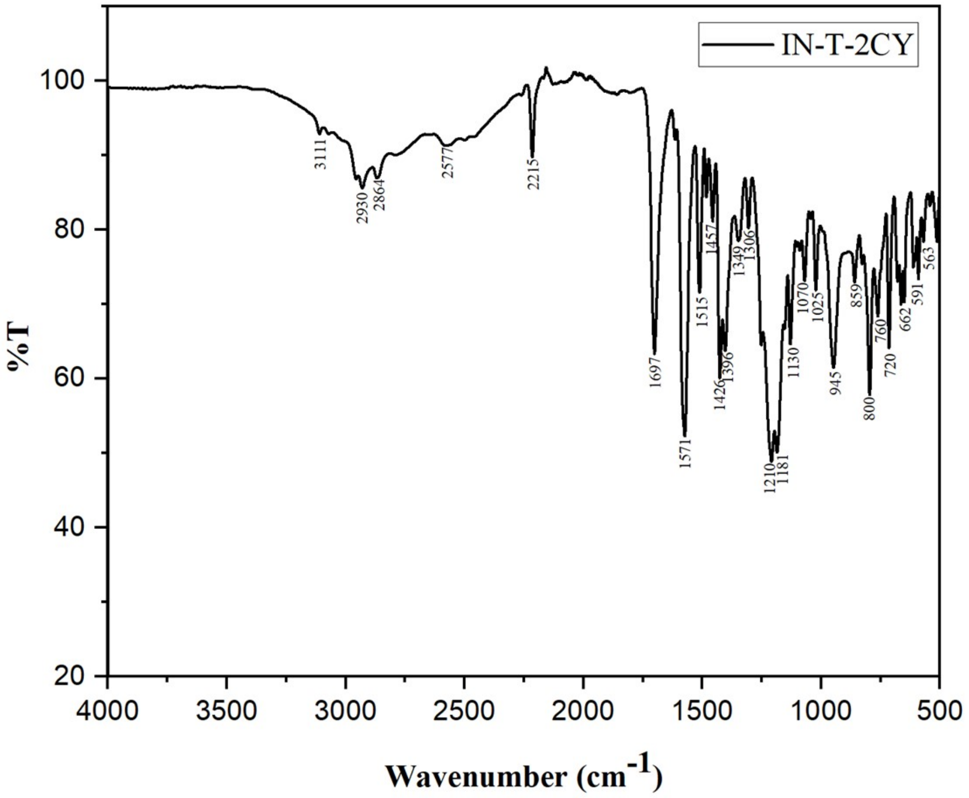

Signature SIF VIT VELLORE
IN-T-2A

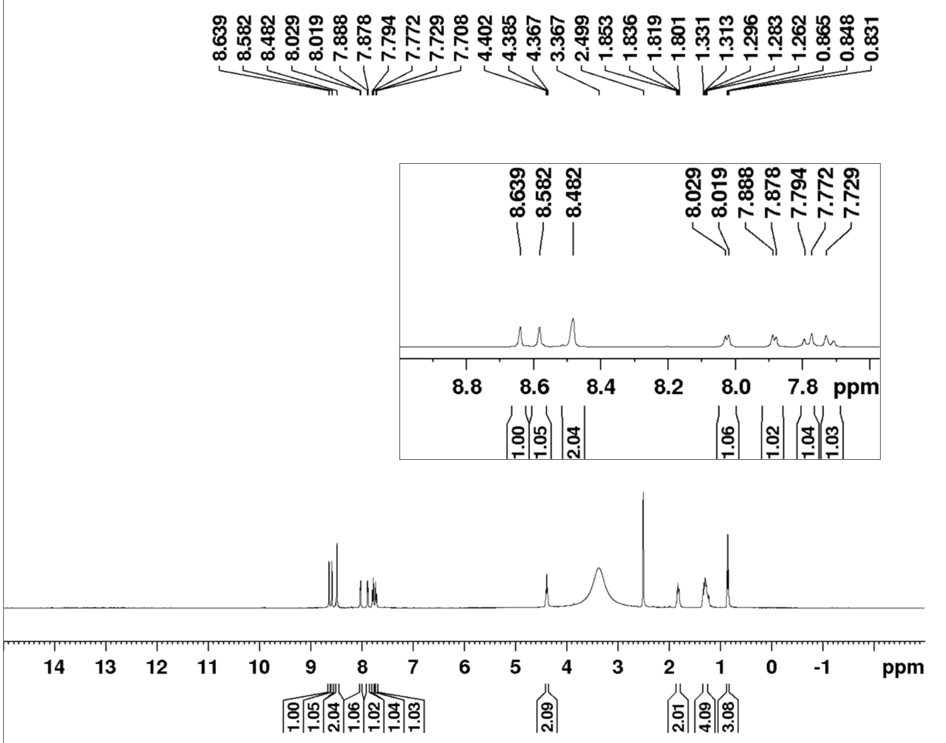

BRUKER

Current Data Parameters
NAME Dr.SLM270323
EXPNO 20
PROCNO 1

F2 - Acquisition Parameters
Date_ 20230327
Time 13.19 h
INSTRUM spect
PROBHD Z108618_0505 (
PULPROG zg30
TD 65536
SOLVENT DMSO
NS 32
DS 2
SWH 8012.820 Hz
FIDRES 0.244532 Hz
AQ 4.0894465 sec
RG 143.73
DW 62.400 usec
DE 6.50 usec
TE 306.6 K
D1 1.00000000 sec
TD0 1
SFO1 400.2604716 MHz
NUC1 1H
P1 15.00 usec
PLW1 14.95499992 W

F2 - Processing parameters
SI 65536
SF 400.2580031 MHz
WDW EM
SSB 0
LB 0.30 Hz
GB 0
PC 1.00

Signature SIF VIT VELLORE
IN-T-2CY

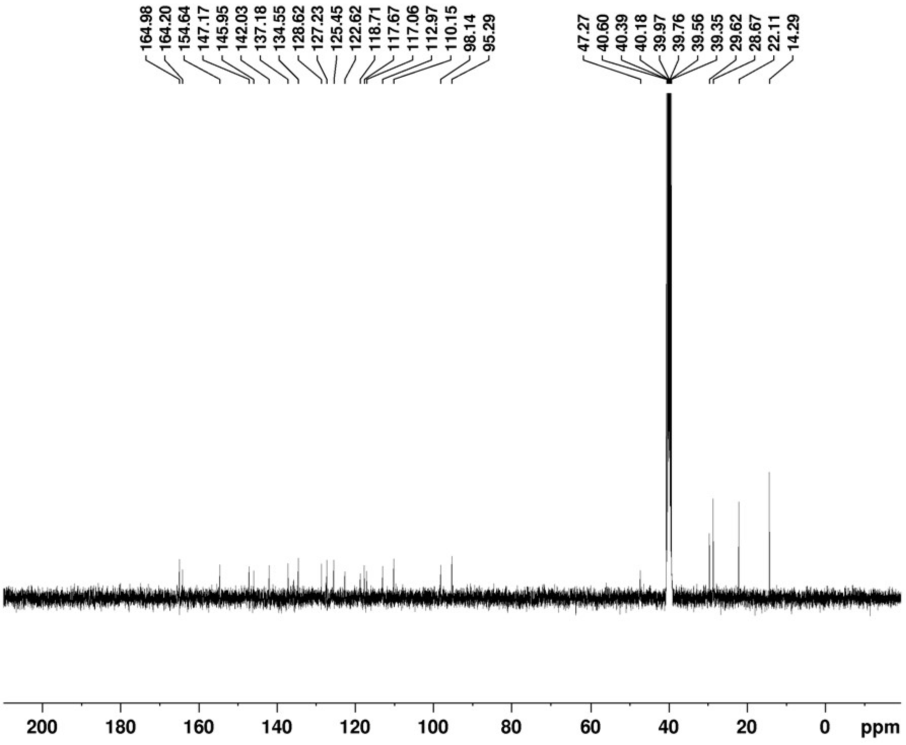

BRUKER

Current Data Parameters
NAME Dr.SLM280323
EXPNO 26
PROCNO 1

F2 - Acquisition Parameters
Date_ 20230329
Time 5.30 h
INSTRUM spect
PROBHD Z108618_0505 (
PULPROG zgpg30
TD 65536
SOLVENT DMSO
NS 512
DS 4
SWH 24038.461 Hz
FIDRES 0.733596 Hz
AQ 1.3631488 sec
RG 199.6
DW 20.800 usec
DE 6.50 usec
TE 304.1 K
D1 2.00000000 sec
D11 0.03000000 sec
TD0 1
SFO1 100.6550186 MHz
NUC1 13C
P1 10.00 usec
PLW1 58.22499847 W
SFO2 400.2596010 MHz
NUC2 1H
CPDPRG[2 waltz16
PCPD2 90.00 usec
PLW2 14.95499992 W
PLW12 0.41542000 W
PLW13 0.20895000 W

F2 - Processing parameters
SI 32768
SF 100.6449542 MHz
WDW EM
SSB 0
LB 1.00 Hz
GB 0
PC 1.40

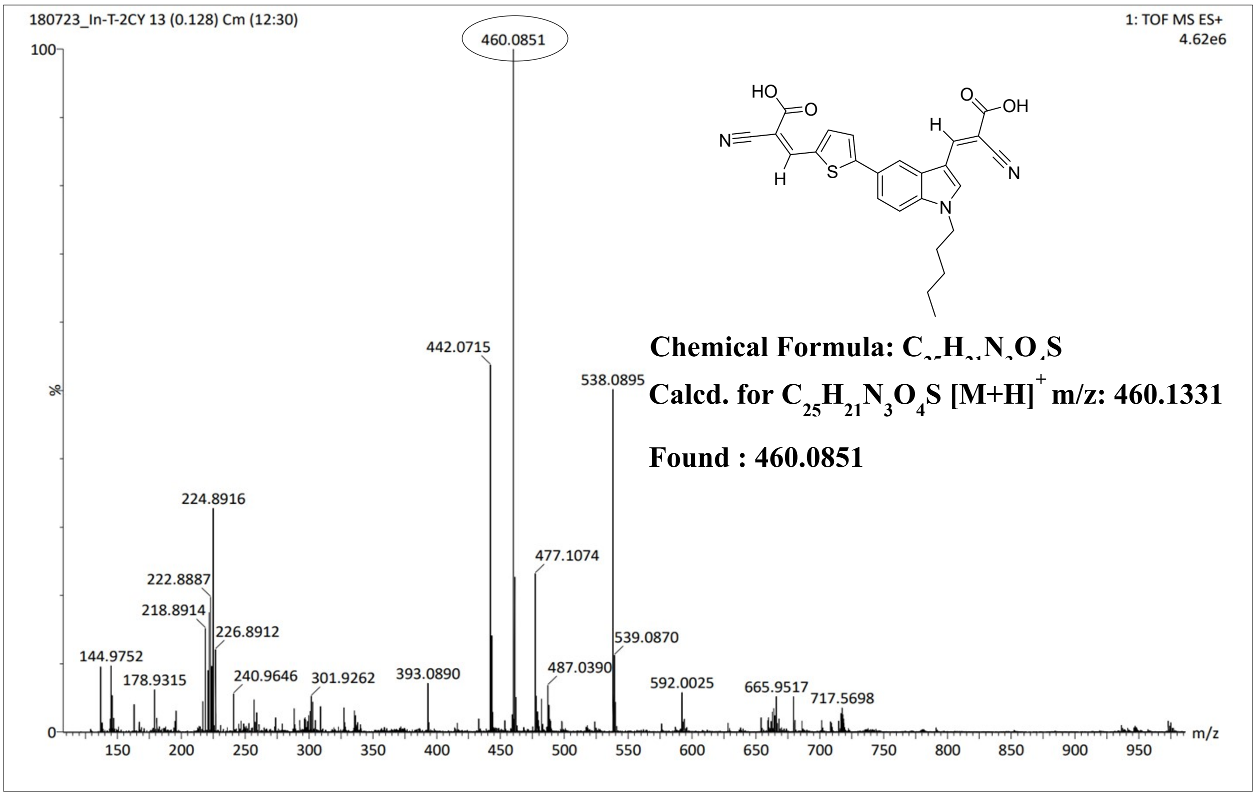

180723_In-T-2CY 13 (0.128) Cm (12:30)
1: TOF MS ES+
4.62e6
100
%
0
460.0851
442.0715
538.0895
224.8916
222.8887
218.8914
226.8912
144.9752
178.9315
240.9646
301.9262
393.0890
477.1074
487.0390
539.0870
592.0025
665.9517
717.5698
150
200
250
300
350
400
450
500
550
600
650
700
750
800
850
900
950
m/z
HO
O
OH
N
H
S
Chemical Formula: C H N O S
Calcd. for $C_{25}H_{21}N_3O_4S$ $[M+H]^+$ m/z: 460.1331
Found : 460.0851

2. 2-cyano-3-(5-(4-cyanophenyl)-1-pentyl-1*H*-indol-3-yl)acrylic acid (In-B-C)

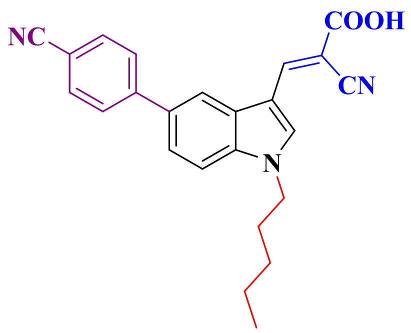

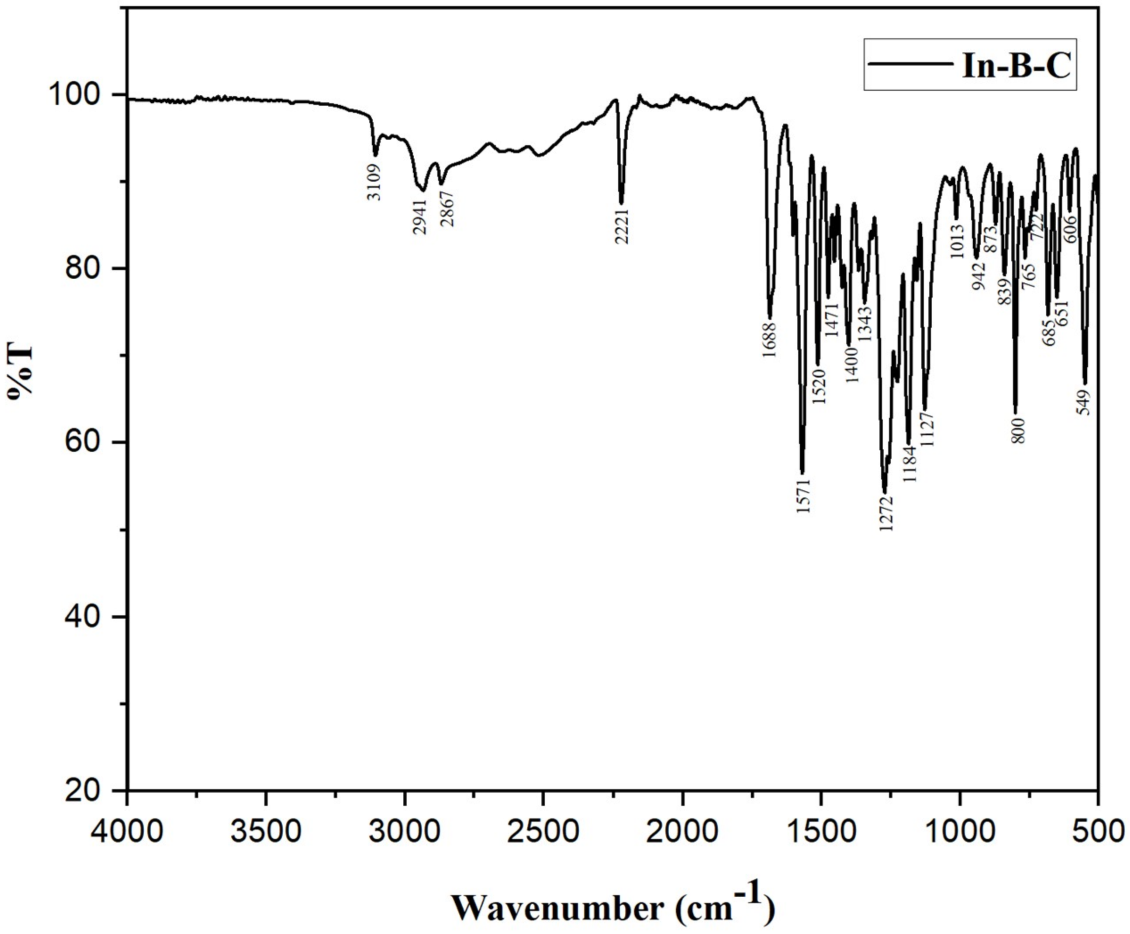

Signature SIF VIT VELLORE
IN-B-CN

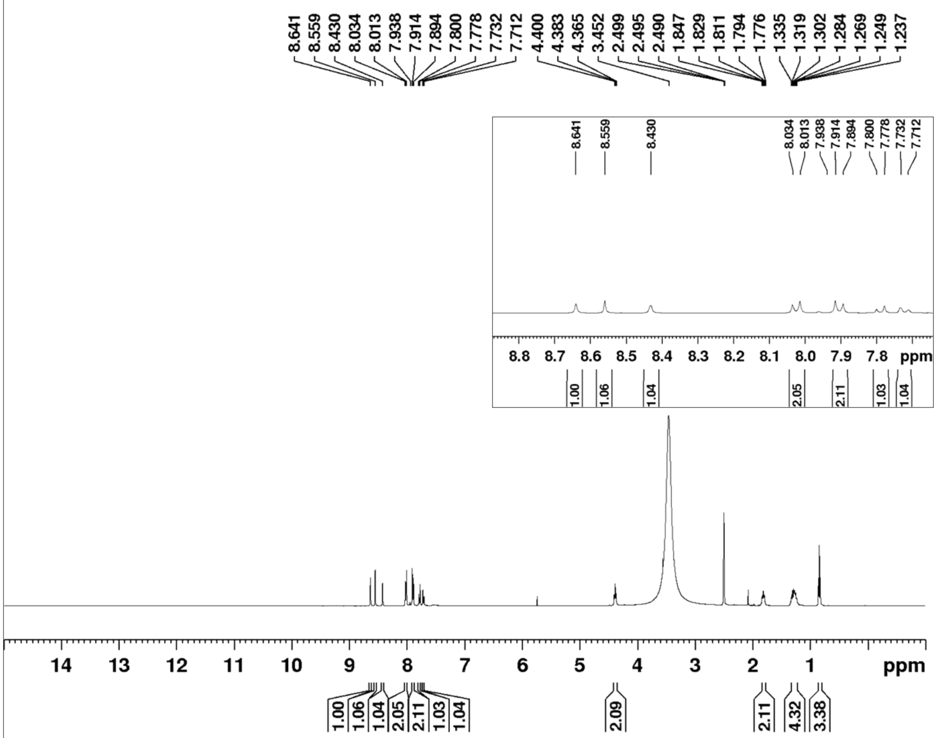

BRUKER

Current Data Parameters
NAME Dr.SLM180723
EXPNO 9
PROCNO 1

F2 - Acquisition Parameters
Date_ 20230718
Time 13.03 h
INSTRUM spect
PROBHD Z108618_0505 (
PULPROG zg30
TD 65536
SOLVENT DMSO
NS 32
DS 2
SWH 8012.820 Hz
FIDRES 0.244532 Hz
AQ 4.0894465 sec
RG 98.85
DW 62.400 usec
DE 6.50 usec
TE 301.6 K
D1 1.00000000 sec
TD0 1
SFO1 400.2604716 MHz
NUC1 1H
P1 15.00 usec
PLW1 14.95499992 W

F2 - Processing parameters
SI 65536
SF 400.2580048 MHz
WDW EM
SSB 0
LB 0.30 Hz
GB 0
PC 1.00

Signature SIF VIT VELLORE
IN-B-C

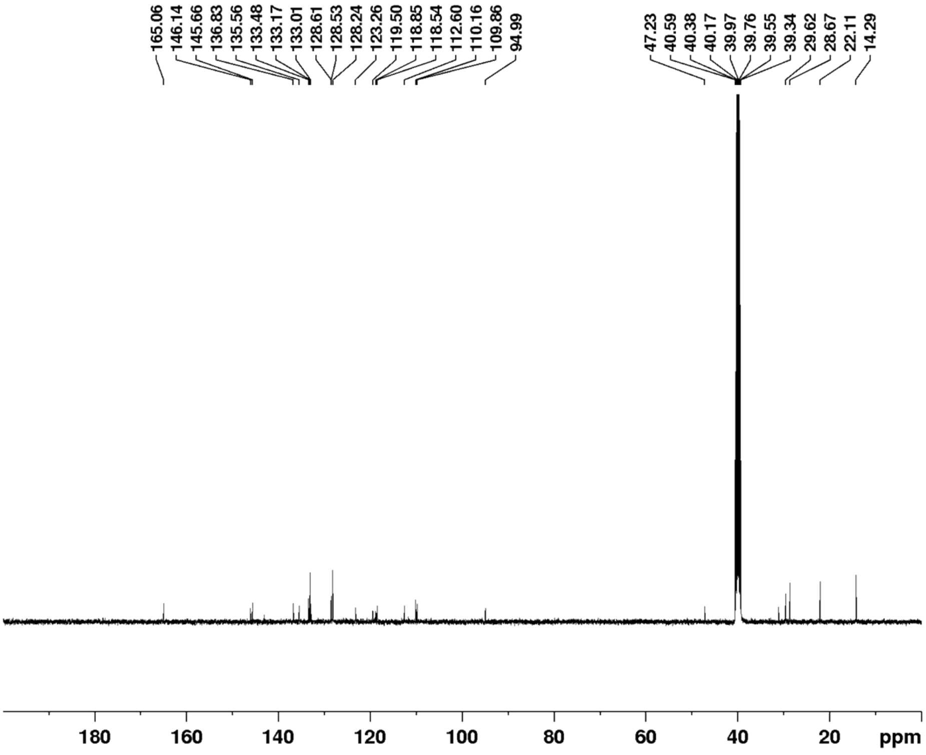

BRUKER

Current Data Parameters
NAME Dr.SLM141223
EXPNO 17
PROCNO 1

F2 - Acquisition Parameters
Date_ 20231215
Time 7.04 h
INSTRUM spect
PROBHD Z108618_0505 (
PULPROG zgpg30
TD 65536
SOLVENT DMSO
NS 512
DS 4
SWH 24038.461 Hz
FIDRES 0.733596 Hz
AQ 1.3631488 sec
RG 199.6
DW 20.800 usec
DE 6.50 usec
TE 303.0 K
D1 2.00000000 sec
D11 0.03000000 sec
TD0 1
SFO1 100.6550186 MHz
NUC1 13C
P1 10.00 usec
PLW1 56.49300003 W
SFO2 400.2596010 MHz
NUC2 1H
CPDPRG[2 waltz16
PCPD2 90.00 usec
PLW2 15.21399975 W
PLW12 0.42261001 W
PLW13 0.21257000 W

F2 - Processing parameters
SI 32768
SF 100.6449542 MHz
WDW EM
SSB 0
LB 1.00 Hz
GB 0
PC 1.40

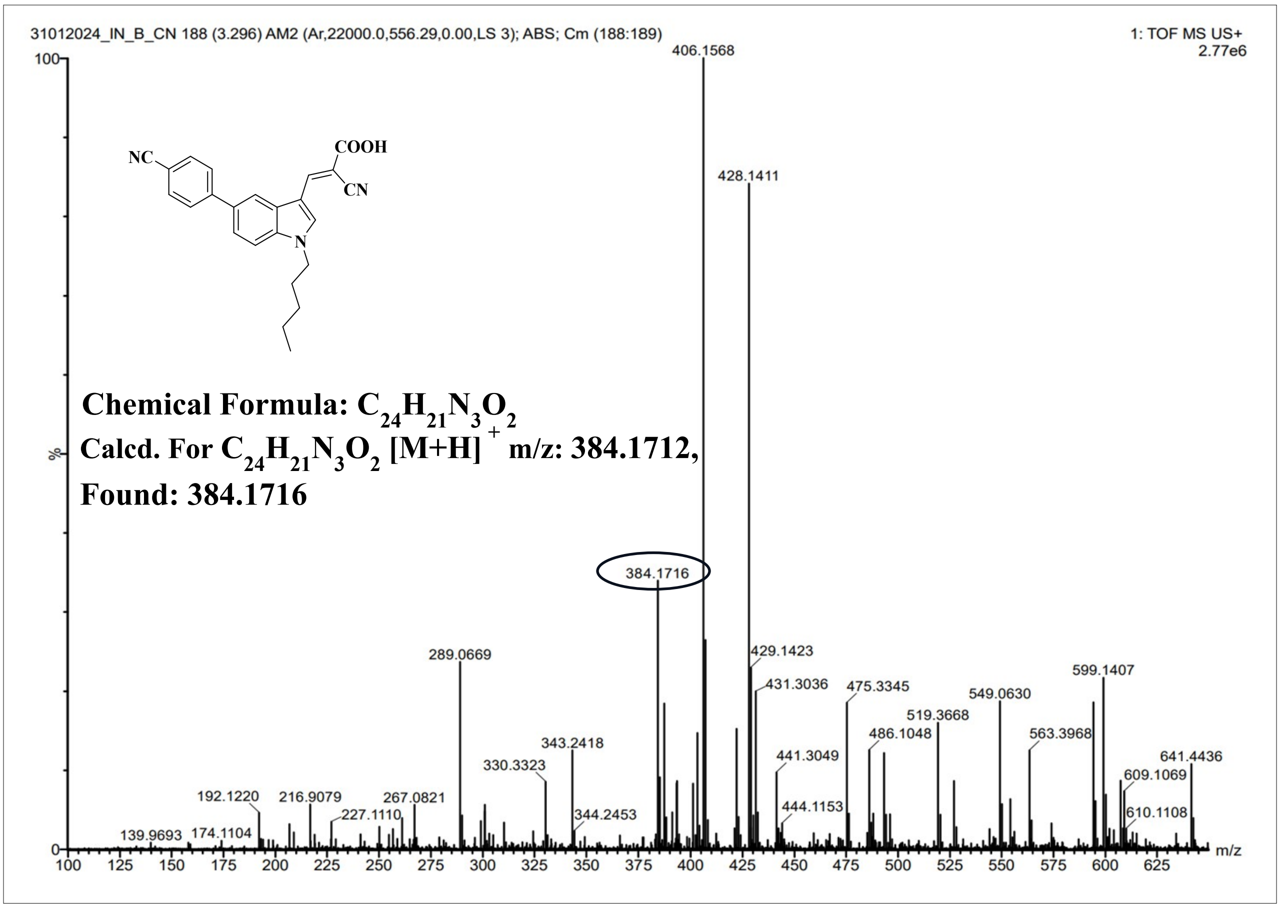
31012024_IN_B_CN 188 (3.296) AM2 (Ar,22000.0,556.29,0.00,LS 3); ABS; Cm (188:189)
1: TOF MS US+
2.77e6
NC
COOH
CN
N
Chemical Formula: $C_{24}H_{21}N_3O_2$
Calcd. For $C_{24}H_{21}N_3O_2$ $[M+H]^+$ m/z: 384.1712,
Found: 384.1716
%
406.1568
428.1411
384.1716
289.0669
429.1423
431.3036
475.3345
549.0630
599.1407
519.3668
486.1048
563.3968
343.2418
441.3049
641.4436
330.3323
609.1069
192.1220
216.9079
267.0821
227.1110
444.1153
610.1108
344.2453
139.9693
174.1104
m/z

3. 5-bromo-1-pentyl-1H-indole-3-carbaldehyde (1a)

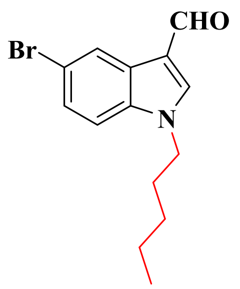

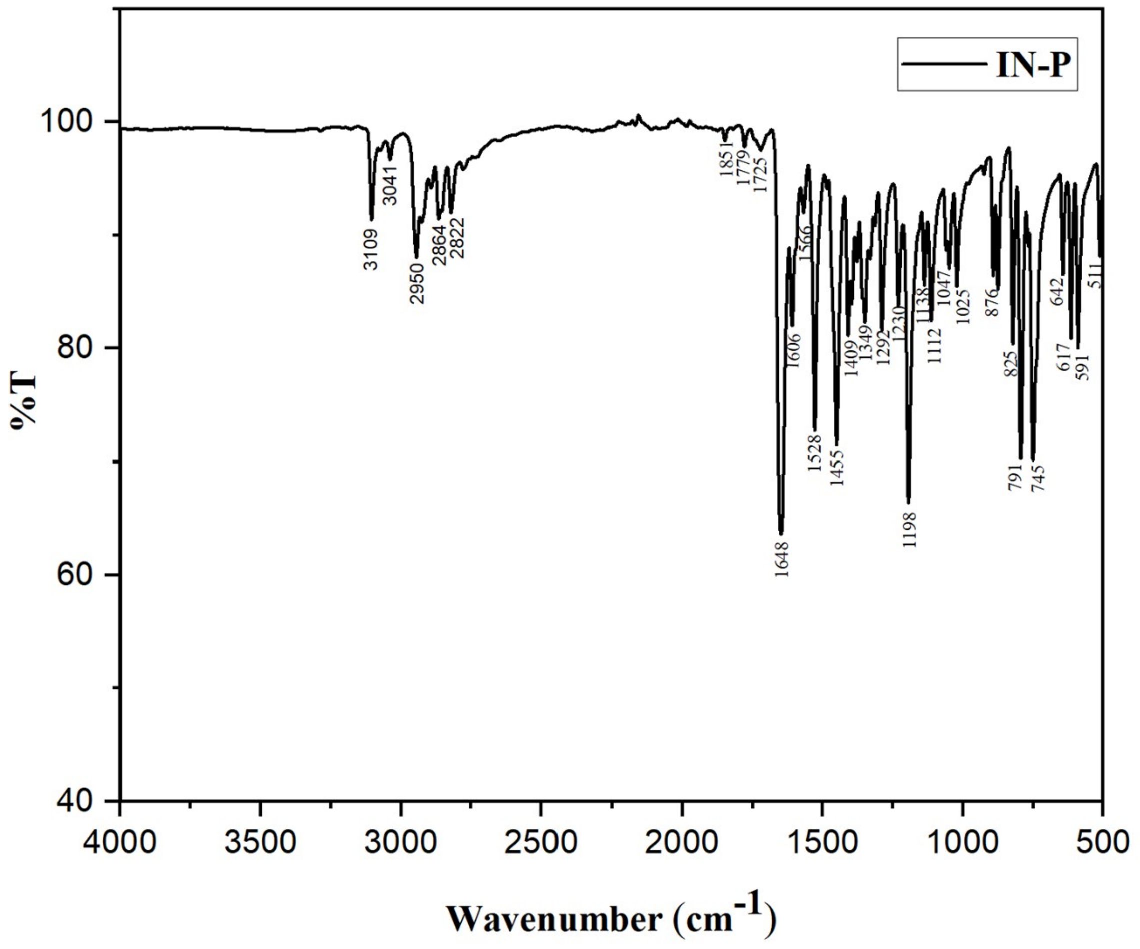

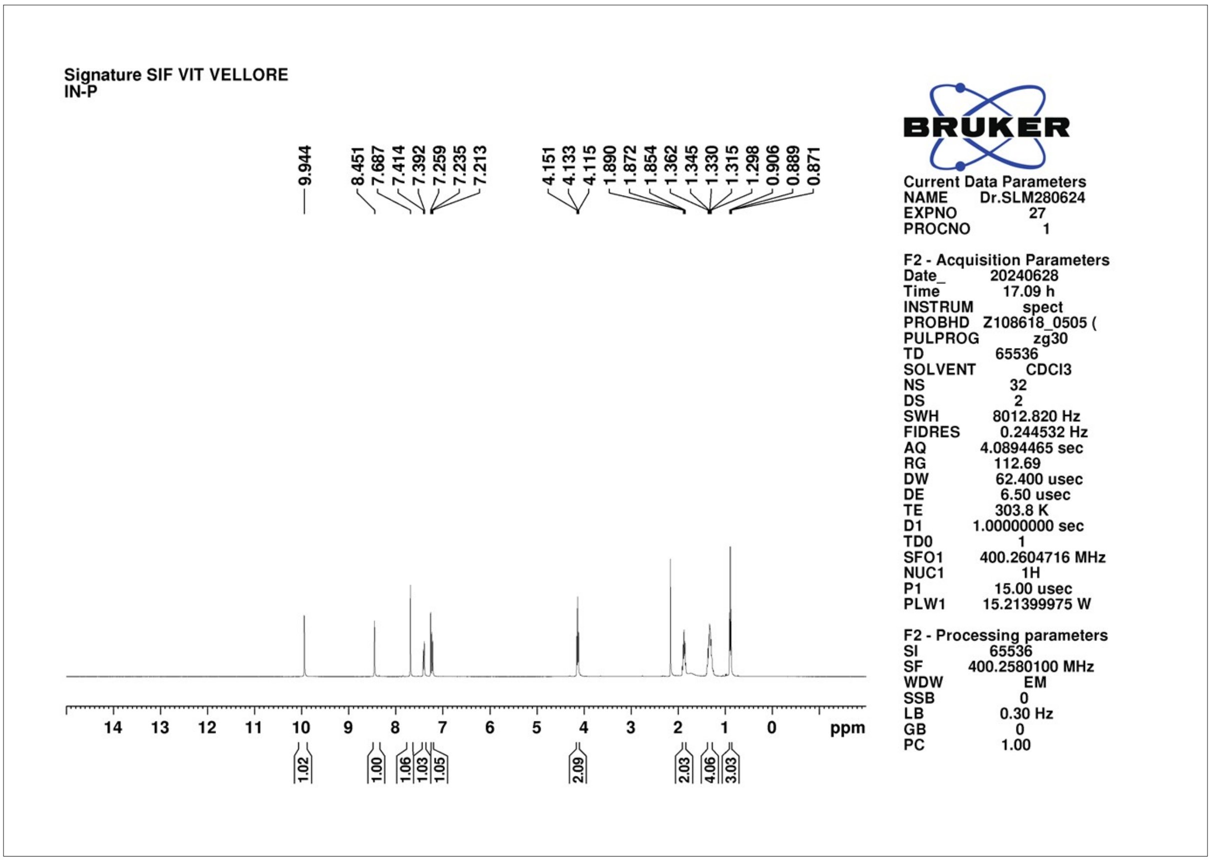
Signature SIF VIT VELLORE
IN-P
9.944
8.451
7.687
7.414
7.392
7.259
7.235
7.213
4.151
4.133
4.115
1.890
1.872
1.854
1.362
1.345
1.330
1.315
1.298
0.906
0.889
0.871
14 13 12 11 10 9 8 7 6 5 4 3 2 1 0 ppm
1.02
1.00
1.06
1.03
1.05
2.09
2.03
4.06
3.03
BRUKER
Current Data Parameters
NAME Dr.SLM280624
EXPNO 27
PROCNO 1
F2 - Acquisition Parameters
Date_ 20240628
Time 17.09 h
INSTRUM spect
PROBHD Z108618_0505 (
PULPROG zg30
TD 65536
SOLVENT CDCl3
NS 32
DS 2
SWH 8012.820 Hz
FIDRES 0.244532 Hz
AQ 4.0894465 sec
RG 112.69
DW 62.400 usec
DE 6.50 usec
TE 303.8 K
D1 1.00000000 sec
TD0 1
SFO1 400.2604716 MHz
NUC1 1H
P1 15.00 usec
PLW1 15.21399975 W
F2 - Processing parameters
SI 65536
SF 400.2580100 MHz
WDW EM
SSB 0
LB 0.30 Hz
GB 0
PC 1.00

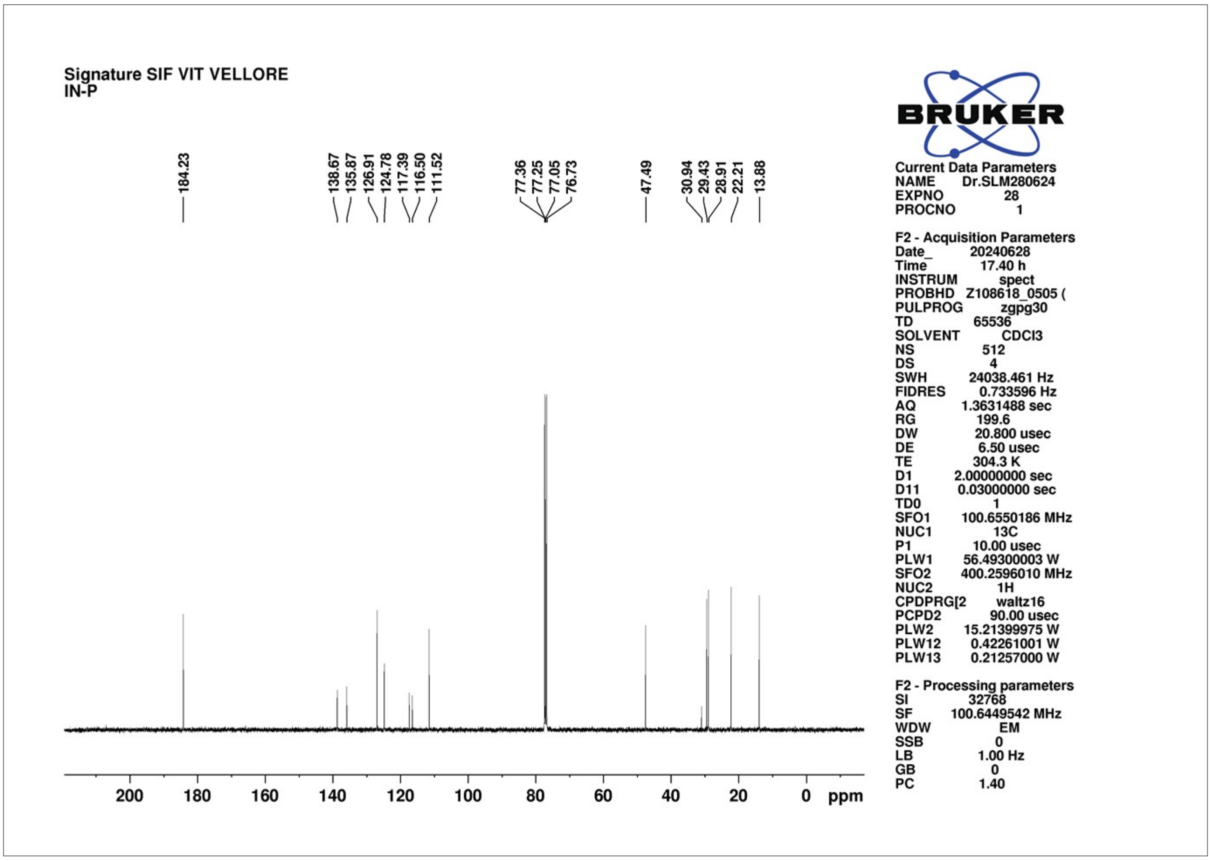
Signature SIF VIT VELLORE
IN-P
184.23
138.67
135.87
126.91
124.78
117.39
116.50
111.52
77.36
77.25
77.05
76.73
47.49
30.94
29.43
28.91
22.21
13.88
200 180 160 140 120 100 80 60 40 20 0 ppm
BRUKER
Current Data Parameters
NAME Dr.SLM280624
EXPNO 28
PROCNO 1
F2 - Acquisition Parameters
Date_ 20240628
Time 17.40 h
INSTRUM spect
PROBHD Z108618_0505 (
PULPROG zgpg30
TD 65536
SOLVENT CDCl3
NS 512
DS 4
SWH 24038.461 Hz
FIDRES 0.733596 Hz
AQ 1.3631488 sec
RG 199.6
DW 20.800 usec
DE 6.50 usec
TE 304.3 K
D1 2.00000000 sec
D11 0.03000000 sec
TD0 1
SFO1 100.6550186 MHz
NUC1 13C
P1 10.00 usec
PLW1 56.49300003 W
SFO2 400.2596010 MHz
NUC2 1H
CPDPRG[2 waltz16
PCPD2 90.00 usec
PLW2 15.21399975 W
PLW12 0.42261001 W
PLW13 0.21257000 W
F2 - Processing parameters
SI 32768
SF 100.6449542 MHz
WDW EM
SSB 0
LB 1.00 Hz
GB 0
PC 1.40

4. 5-(5-formylthiophen-2-yl)-1-pentyl-1*H*-indole-3-carbaldehyde (2b)

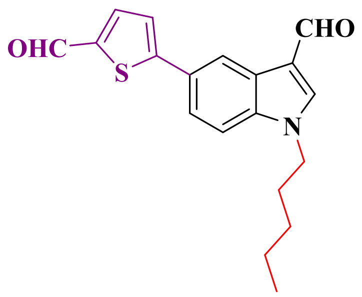

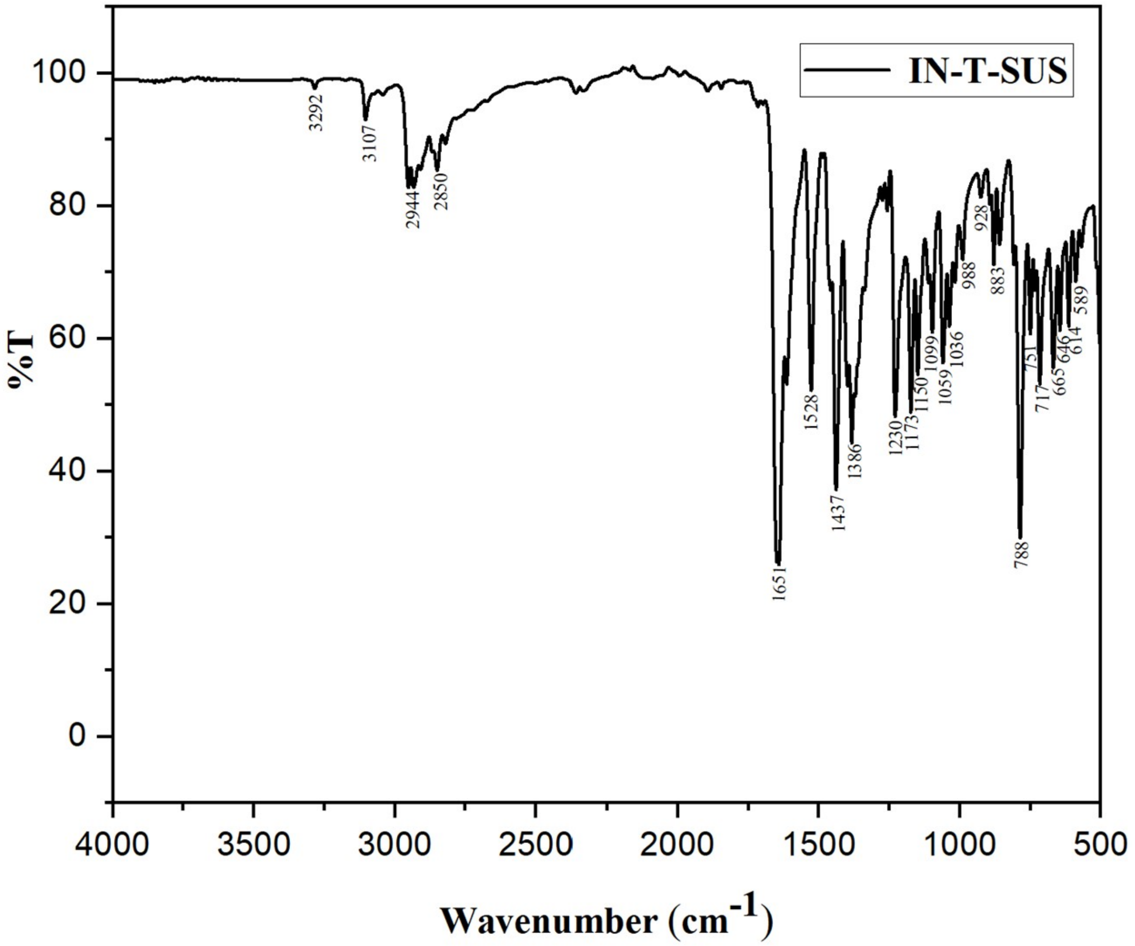

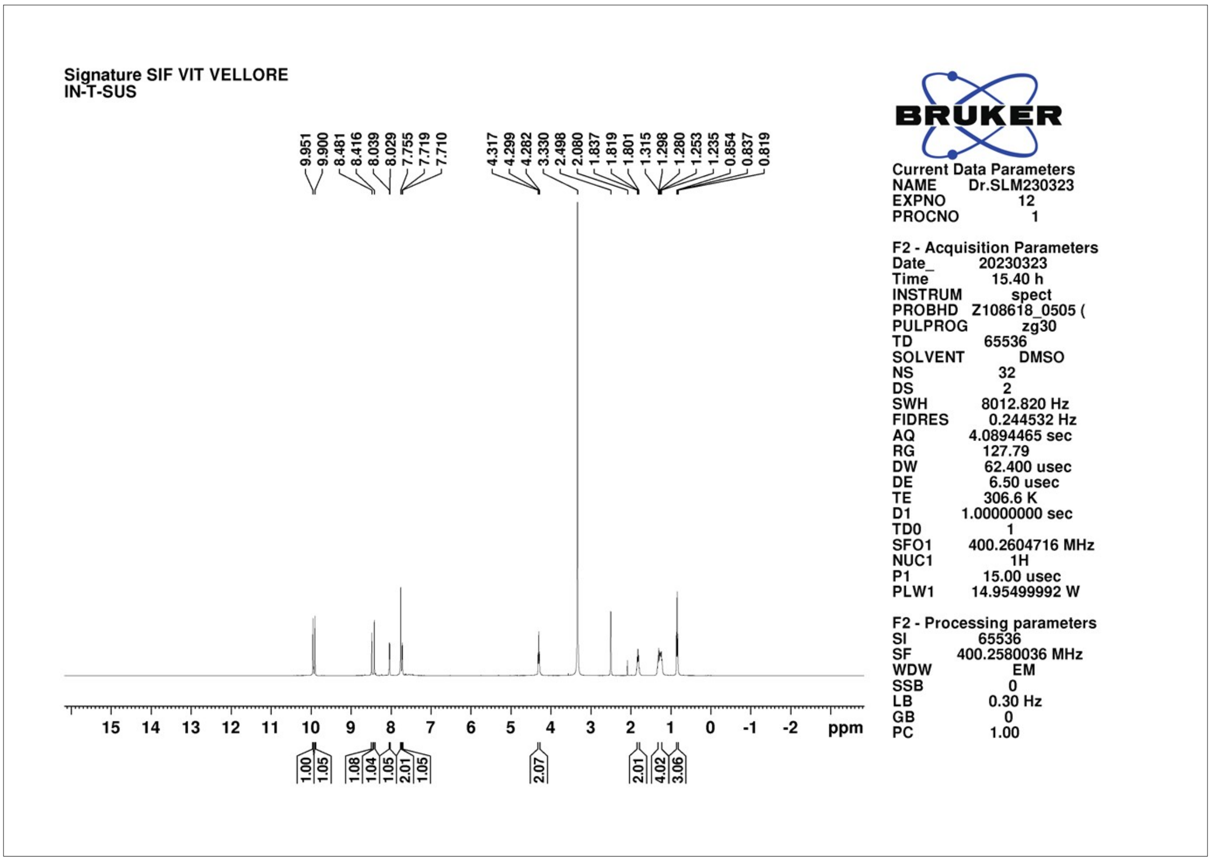

Signature SIF VIT VELLORE
IN-T-SUS
9.951
9.900
8.481
8.416
8.039
8.029
7.755
7.719
7.710
4.317
4.299
4.282
3.330
2.498
2.080
1.837
1.819
1.801
1.315
1.298
1.280
1.253
1.235
0.854
0.837
0.819
15 14 13 12 11 10 9 8 7 6 5 4 3 2 1 0 -1 -2 ppm
1.00
1.05
1.08
1.04
1.05
2.01
1.05
2.07
2.01
4.02
3.06
BRUKER
Current Data Parameters
NAME Dr.SLM230323
EXPNO 12
PROCNO 1
F2 - Acquisition Parameters
Date_ 20230323
Time 15.40 h
INSTRUM spect
PROBHD Z108618_0505 (
PULPROG zg30
TD 65536
SOLVENT DMSO
NS 32
DS 2
SWH 8012.820 Hz
FIDRES 0.244532 Hz
AQ 4.0894465 sec
RG 127.79
DW 62.400 usec
DE 6.50 usec
TE 306.6 K
D1 1.00000000 sec
TD0 1
SFO1 400.2604716 MHz
NUC1 1H
P1 15.00 usec
PLW1 14.95499992 W
F2 - Processing parameters
SI 65536
SF 400.2580036 MHz
WDW EM
SSB 0
LB 0.30 Hz
GB 0
PC 1.00

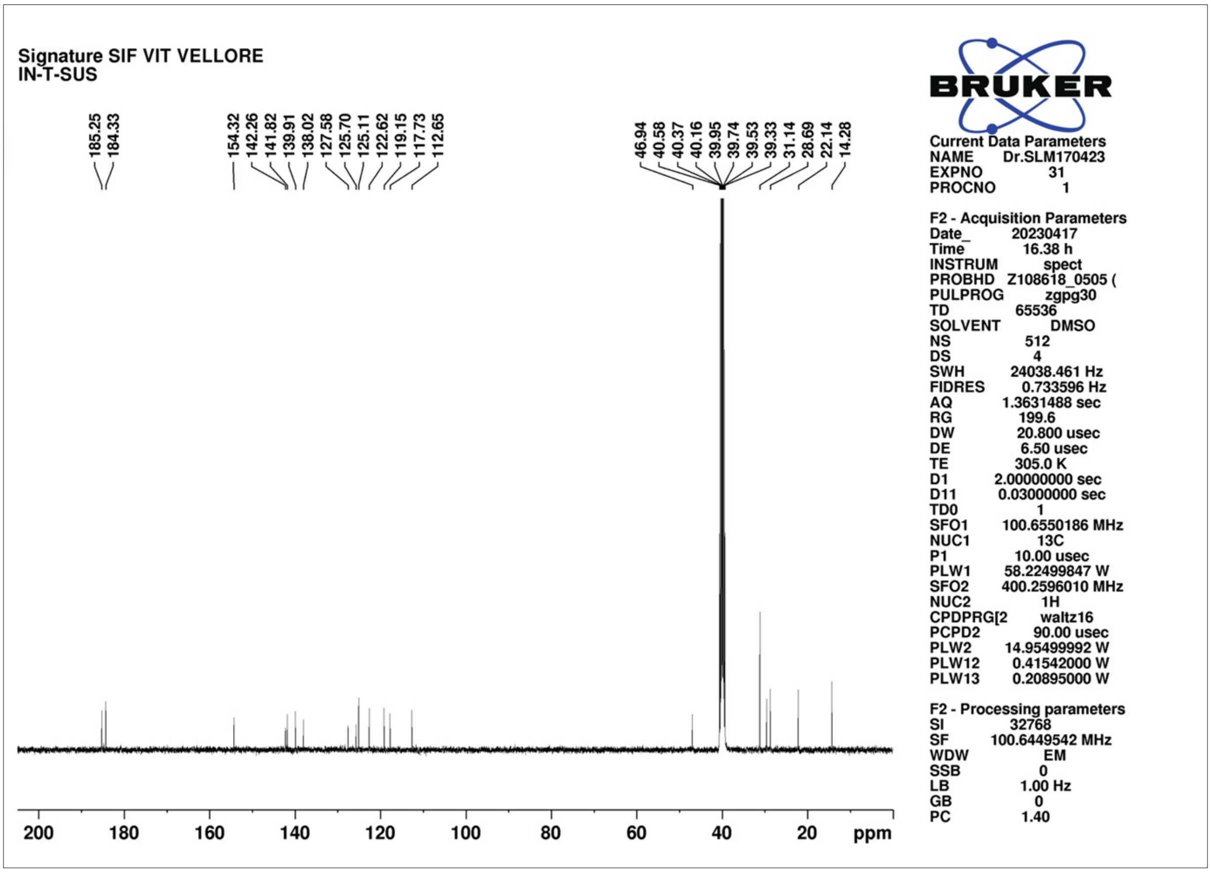

Signature SIF VIT VELLORE
IN-T-SUS
185.25
184.33
154.32
142.26
141.82
139.91
138.02
127.58
125.70
125.11
122.62
119.15
117.73
112.65
46.94
40.58
40.37
40.16
39.95
39.74
39.53
39.33
31.14
28.69
22.14
14.28
200 180 160 140 120 100 80 60 40 20 ppm
BRUKER
Current Data Parameters
NAME Dr.SLM170423
EXPNO 31
PROCNO 1
F2 - Acquisition Parameters
Date_ 20230417
Time 16.38 h
INSTRUM spect
PROBHD Z108618_0505 (
PULPROG zgpg30
TD 65536
SOLVENT DMSO
NS 512
DS 4
SWH 24038.461 Hz
FIDRES 0.733596 Hz
AQ 1.3631488 sec
RG 199.6
DW 20.800 usec
DE 6.50 usec
TE 305.0 K
D1 2.00000000 sec
D11 0.03000000 sec
TD0 1
SFO1 100.6550186 MHz
NUC1 13C
P1 10.00 usec
PLW1 58.22499847 W
SFO2 400.2596010 MHz
NUC2 1H
CPDPRG[2 waltz16
PCPD2 90.00 usec
PLW2 14.95499992 W
PLW12 0.41542000 W
PLW13 0.20895000 W
F2 - Processing parameters
SI 32768
SF 100.6449542 MHz
WDW EM
SSB 0
LB 1.00 Hz
GB 0
PC 1.40

5. 4-(3-formyl-1-pentyl-1*H*-indol-5-yl)benzonitrile (2c)

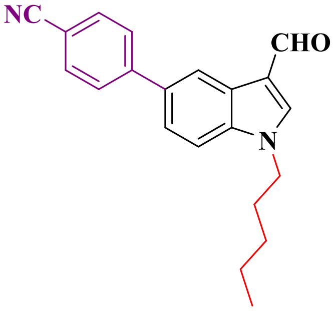

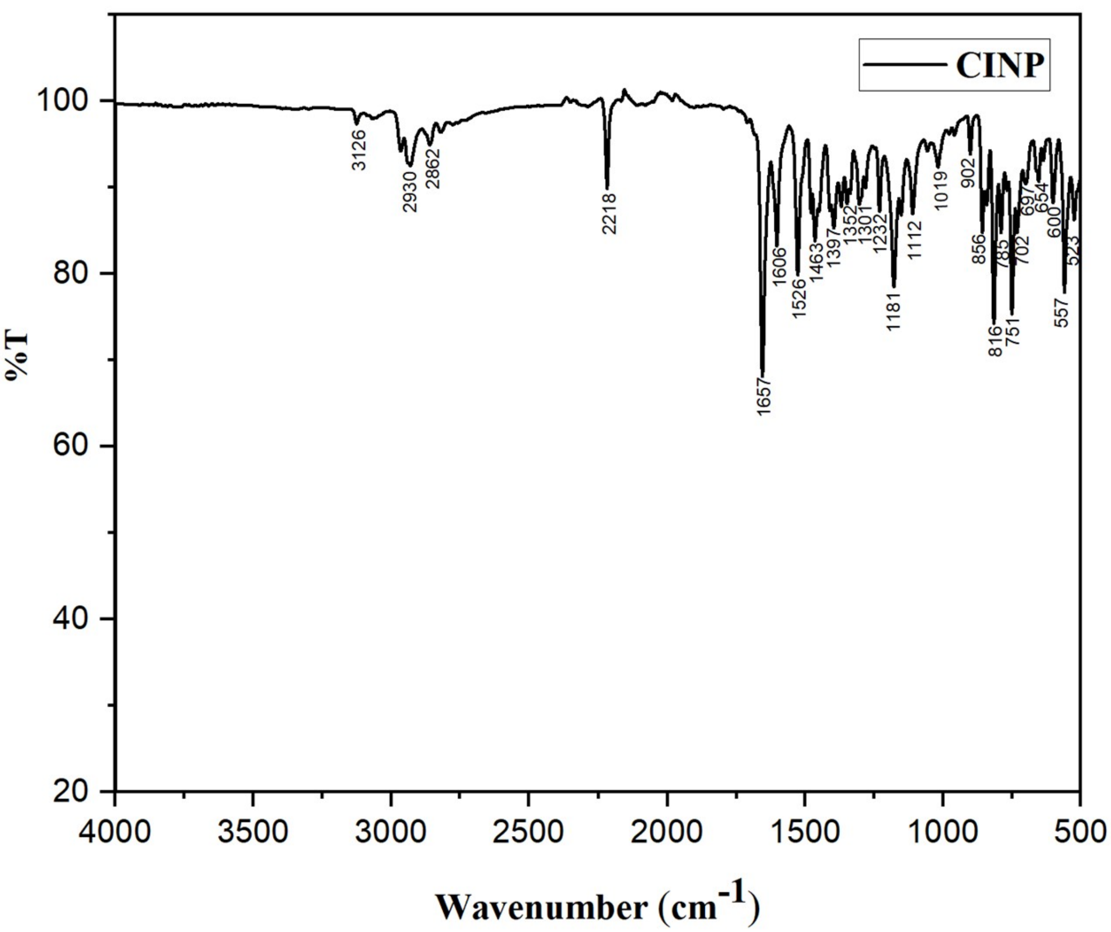

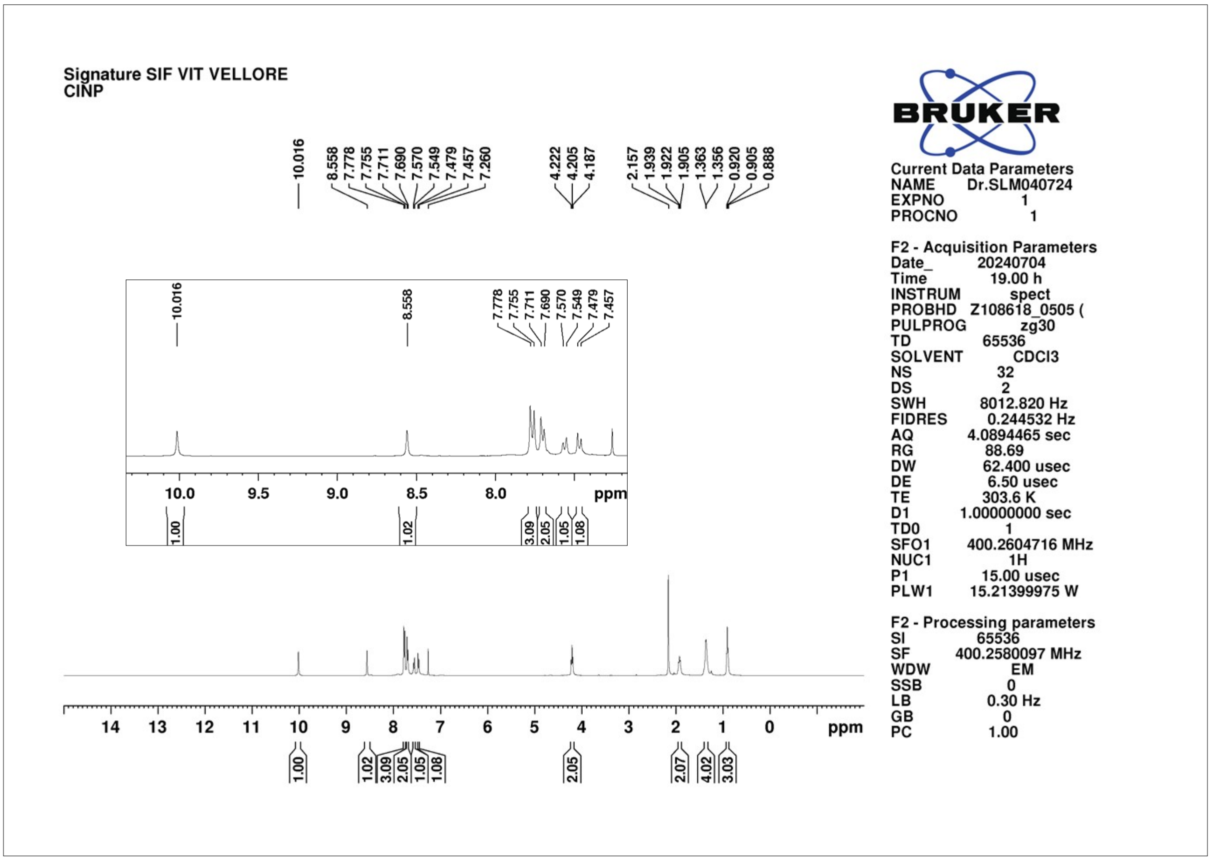
Signature SIF VIT VELLORE
CINP
10.016
8.558
7.778
7.755
7.711
7.690
7.570
7.549
7.479
7.457
7.260
4.222
4.205
4.187
2.157
1.939
1.922
1.905
1.363
1.356
0.920
0.905
0.888
1.00
1.02
3.09
2.05
1.05
1.08
2.05
2.07
4.02
3.03
ppm
BRUKER
Current Data Parameters
NAME Dr.SLM040724
EXPNO 1
PROCNO 1
F2 - Acquisition Parameters
Date_ 20240704
Time 19.00 h
INSTRUM spect
PROBHD Z108618_0505 (
PULPROG zg30
TD 65536
SOLVENT CDCl3
NS 32
DS 2
SWH 8012.820 Hz
FIDRES 0.244532 Hz
AQ 4.0894465 sec
RG 88.69
DW 62.400 usec
DE 6.50 usec
TE 303.6 K
D1 1.00000000 sec
TD0 1
SFO1 400.2604716 MHz
NUC1 1H
P1 15.00 usec
PLW1 15.21399975 W
F2 - Processing parameters
SI 65536
SF 400.2580097 MHz
WDW EM
SSB 0
LB 0.30 Hz
GB 0
PC 1.00

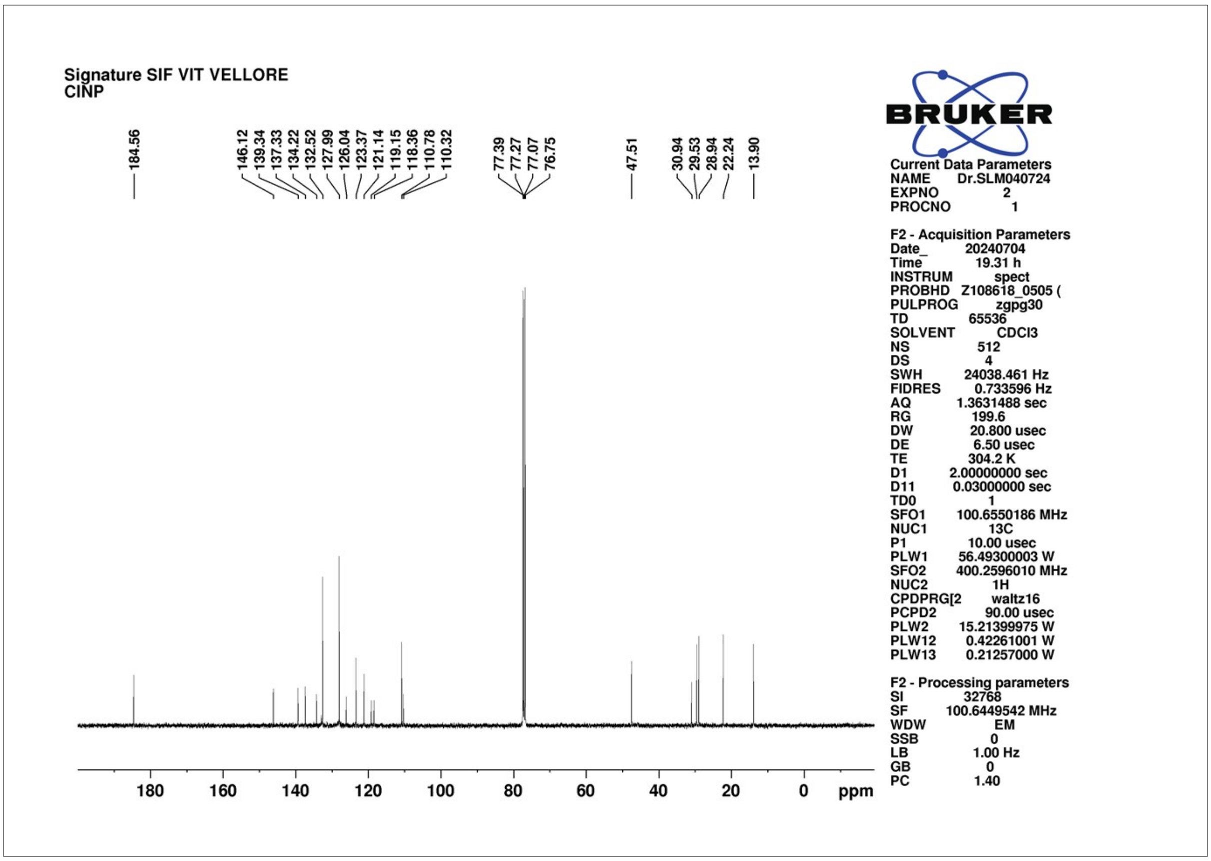
Signature SIF VIT VELLORE
CINP
184.56
146.12
139.34
137.33
134.22
132.52
127.99
126.04
123.37
121.14
119.15
118.36
110.78
110.32
77.39
77.27
77.07
76.75
47.51
30.94
29.53
28.94
22.24
13.90
ppm
BRUKER
Current Data Parameters
NAME Dr.SLM040724
EXPNO 2
PROCNO 1
F2 - Acquisition Parameters
Date_ 20240704
Time 19.31 h
INSTRUM spect
PROBHD Z108618_0505 (
PULPROG zgpg30
TD 65536
SOLVENT CDCl3
NS 512
DS 4
SWH 24038.461 Hz
FIDRES 0.733596 Hz
AQ 1.3631488 sec
RG 199.6
DW 20.800 usec
DE 6.50 usec
TE 304.2 K
D1 2.00000000 sec
D11 0.03000000 sec
TD0 1
SFO1 100.6550186 MHz
NUC1 13C
P1 10.00 usec
PLW1 56.49300003 W
SFO2 400.2596010 MHz
NUC2 1H
CPDPRG[2 waltz16
PCPD2 90.00 usec
PLW2 15.21399975 W
PLW12 0.42261001 W
PLW13 0.21257000 W
F2 - Processing parameters
SI 32768
SF 100.6449542 MHz
WDW EM
SSB 0
LB 1.00 Hz
GB 0
PC 1.40

Crystallographic data of 3-(5-(3-((2-carboxy-2-cyanovinyl)-1-pentyl-1*H*-indol-5-yl)thiophen-2-yl)-2-isocyanoacrylic acid (In-T-2C)

InT2CY_030723_0m

Table 1 Crystal data and structure refinement for InT2CY_030723_0m.

Identification code	InT2CY_030723_0m
Empirical formula	$C_{25}H_{21}N_3O_4S$
Formula weight	459.5200
Temperature/K	300.00
Crystal system	triclinic
Space group	P-1
a/Å	10.4665(7)
b/Å	10.8561(8)
c/Å	16.2199(11)
α/°	86.643(3)
β/°	76.578(2)
γ/°	61.751(2)
Volume/Å^3	1576.31(19)
Z	4
ρ_{calc}g/cm^3	0.968
μ/mm^{-1}	0.130
F(000)	480.6
Crystal size/mm^3	0.234 × 0.179 × 0.087
Radiation	Mo Kα (λ = 0.71073)
2Θ range for data collection/°	4.54 to 50.12
Index ranges	$-12 \leq h \leq 12$, $-12 \leq k \leq 12$, $-19 \leq l \leq 19$
Reflections collected	38218
Independent reflections	5535 [R_{int} = 0.0660, R_{sigma} = 0.0500]
Data/restraints/parameters	5535/2/299
Goodness-of-fit on F^2	1.224
Final R indexes [I>=2σ (I)]	R_1 = 0.0873, wR_2 = 0.2923
Final R indexes [all data]	R_1 = 0.1375, wR_2 = 0.3620
Largest diff. peak/hole / e Å^{-3}	0.42/-0.47

Table 2 Fractional Atomic Coordinates (×10^4) and Equivalent Isotropic Displacement Parameters (Å^2×10^3) for InT2CY_030723_0m. U_{eq} is defined as 1/3 of the trace of the orthogonalised U_{IJ} tensor.

Atom	*x*	*y*	*z*	U(eq)
S2	5388.3(14)	1915.0(13)	4213.6(8)	71.3(4)
O2	11313(4)	613(4)	3107(3)	94.6(11)
C21	2927(5)	2134(4)	5426(3)	61.9(12)
N20	-1560(4)	3235(4)	5959(3)	68.3(10)
C1	-517(5)	4152(4)	3809(3)	65.2(12)
C3	4508(5)	1755(4)	5219(3)	61.0(11)
C	5493(5)	1219(5)	5733(3)	68.9(13)
C4	2256(6)	1898(5)	6256(3)	67.0(12)
C5	541(5)	3063(4)	5079(3)	59.0(11)
C6	-67(5)	2804(4)	5905(3)	62.2(12)
O4	168(5)	4963(4)	2190(2)	100.5(12)
C7	-1536(6)	4746(5)	3331(3)	69.2(13)
C2	8430(5)	1123(4)	3947(3)	67.6(13)
C8	2051(5)	2708(4)	4854(3)	59.2(11)
C9	-681(5)	3692(5)	4653(3)	64.0(12)
C10	801(6)	2228(5)	6493(3)	68.1(12)
O	-2237(5)	5994(4)	2142(3)	103.4(12)
C11	6943(6)	993(5)	5332(3)	76.0(14)
O3	10627(4)	1017(5)	1881(3)	108.2(13)
C12	-2580(6)	3136(6)	6736(3)	80.8(15)
C13	8825(5)	1236(5)	3089(3)	72.9(13)
C14	10353(6)	950(5)	2682(4)	81.1(15)
C15	7077(5)	1325(5)	4485(3)	65.2(12)
C16	-1916(5)	3771(5)	5227(3)	65.5(12)
C17	-3039(7)	4958(5)	3638(3)	73.7(14)
C18	7789(7)	1552(6)	2565(4)	88.3(16)
C19	-1203(7)	5273(6)	2487(3)	80.0(15)
N	-4223(6)	5123(5)	3889(3)	99.9(15)
C1a	-3219(6)	4356(6)	7355(3)	90.3(16)
N1c	7018(6)	1784(7)	2129(4)	129(2)
C1d	-4311(10)	4243(9)	8106(5)	139(3)
C1e	-3702(15)	3139(11)	8666(8)	226(6)
C1g	-4868(15)	3175(12)	9472(6)	227(6)

Table 3 Anisotropic Displacement Parameters (Å^2×10^3) for InT2CY_030723_0m. The Anisotropic displacement factor exponent takes the form: $-2\pi^2[h^2a^{*2}U_{11}+2hka^*b^*U_{12}+\ldots]$.

Atom	U_{11}	U_{22}	U_{33}	U_{12}	U_{13}	U_{23}
S2	65.2(8)	76.4(9)	76.3(9)	-34.1(7)	-24.9(7)	15.3(6)
O2	63(2)	123(3)	98(3)	-40(2)	-31(2)	21(2)
C21	65(3)	56(3)	66(3)	-28(2)	-18(2)	1(2)
N20	66(3)	69(2)	74(3)	-36(2)	-17(2)	7(2)
C1	62(3)	59(3)	69(3)	-24(2)	-16(2)	2(2)
C3	70(3)	56(3)	61(3)	-32(2)	-16(2)	1(2)
C	65(3)	78(3)	66(3)	-33(3)	-25(3)	14(2)
C4	73(3)	62(3)	65(3)	-28(3)	-24(3)	12(2)
C5	61(3)	50(2)	65(3)	-26(2)	-14(2)	4(2)
C6	56(3)	58(3)	71(3)	-24(2)	-19(2)	1(2)
O4	89(3)	123(3)	79(3)	-41(2)	-26(2)	15(2)
C7	77(3)	61(3)	69(3)	-28(2)	-28(3)	6(2)
C2	67(3)	58(3)	80(4)	-28(2)	-29(3)	11(2)
C8	57(3)	55(2)	66(3)	-26(2)	-16(2)	5(2)
C9	67(3)	56(3)	66(3)	-27(2)	-18(3)	2(2)
C10	71(3)	68(3)	62(3)	-30(3)	-17(3)	12(2)
O	111(3)	122(3)	97(3)	-61(3)	-54(3)	42(2)
C11	81(4)	72(3)	83(4)	-37(3)	-34(3)	11(3)
O3	88(3)	162(4)	82(3)	-67(3)	-20(2)	21(3)
C12	66(3)	97(4)	84(3)	-48(3)	-5(3)	6(3)
C13	68(3)	72(3)	80(4)	-29(3)	-26(3)	6(3)
C14	77(4)	78(3)	86(4)	-38(3)	-15(3)	7(3)
C15	68(3)	58(3)	82(3)	-37(2)	-25(3)	7(2)
C16	62(3)	63(3)	76(3)	-31(2)	-20(3)	4(2)
C17	74(4)	66(3)	86(4)	-31(3)	-32(3)	11(2)
C18	88(4)	102(4)	78(4)	-49(3)	-19(3)	13(3)
C19	80(4)	94(4)	75(4)	-47(3)	-24(3)	6(3)
N	75(3)	116(4)	119(4)	-50(3)	-33(3)	14(3)
C1a	86(4)	107(4)	76(4)	-47(3)	-8(3)	-4(3)
N1c	94(4)	203(7)	104(4)	-77(4)	-41(3)	23(4)
C1d	147(7)	151(7)	104(5)	-71(6)	1(5)	-4(5)
C1e	282(15)	158(9)	177(10)	-90(9)	18(10)	47(8)
C1g	325(16)	223(11)	123(8)	-155(11)	16(10)	23(8)

Table 4 Bond Lengths for InT2CY_030723_0m.

Atom	Atom	Length/Å	Atom	Atom	Length/Å
S2	C3	1.721(5)	O4	C19	1.283(6)
S2	C15	1.727(5)	C7	C17	1.444(8)
O2	C14	1.250(7)	C7	C19	1.484(7)
C21	C3	1.461(6)	C2	C13	1.372(7)
C21	C4	1.437(7)	C2	C15	1.404(7)
C21	C8	1.370(6)	C9	C16	1.375(7)
N20	C6	1.384(6)	O	C19	1.234(6)
N20	C12	1.485(6)	C11	C15	1.393(7)
N20	C16	1.345(6)	O3	C14	1.269(7)
C1	C7	1.352(6)	C12	C1a	1.489(7)
C1	C9	1.430(7)	C13	C14	1.468(7)
C3	C	1.368(6)	C13	C18	1.435(8)
C	C11	1.412(7)	C17	N	1.139(6)
C4	C10	1.351(7)	C18	N1c	1.126(7)
C5	C6	1.418(6)	C1a	C1d	1.508(9)
C5	C8	1.397(6)	C1d	C1e	1.444(13)
C5	C9	1.451(6)	C1e	C1g	1.557(14)
C6	C10	1.386(6)			

Table 5 Bond Angles for InT2CY_030723_0m.

Atom	Atom	Atom	Angle/°	Atom	Atom	Atom	Angle/°
C15	S2	C3	93.0(2)	C5	C9	C1	122.8(4)
C4	C21	C3	119.3(4)	C16	C9	C1	130.7(5)
C8	C21	C3	122.4(4)	C16	C9	C5	106.5(4)
C8	C21	C4	118.3(4)	C6	C10	C4	118.3(5)
C12	N20	C6	123.2(4)	C15	C11	C	112.8(5)
C16	N20	C6	109.8(4)	C1a	C12	N20	112.6(4)
C16	N20	C12	126.9(4)	C14	C13	C2	120.2(5)
C9	C1	C7	129.7(5)	C18	C13	C2	121.1(5)
C21	C3	S2	121.0(4)	C18	C13	C14	118.6(5)
C	C3	S2	110.6(4)	O3	C14	O2	123.5(5)
C	C3	C21	128.4(4)	C13	C14	O2	121.0(5)
C11	C	C3	113.7(5)	C13	C14	O3	115.5(5)
C10	C4	C21	122.6(4)	C2	C15	S2	127.1(4)
C8	C5	C6	119.0(4)	C11	C15	S2	109.9(4)
C9	C5	C6	106.1(4)	C11	C15	C2	123.0(5)
C9	C5	C8	134.9(4)	C9	C16	N20	110.3(4)
C5	C6	N20	107.3(4)	N	C17	C7	179.3(6)
C10	C6	N20	131.4(5)	N1c	C18	C13	177.5(6)
C10	C6	C5	121.3(4)	C7	C19	O4	115.4(5)
C17	C7	C1	121.2(5)	O	C19	O4	125.5(5)
C19	C7	C1	122.5(5)	O	C19	C7	119.1(5)
C19	C7	C17	116.3(5)	C1d	C1a	C12	110.3(5)
C15	C2	C13	131.7(5)	C1e	C1d	C1a	116.8(8)
C5	C8	C21	120.5(4)	C1g	C1e	C1d	114.0(10)

Table 6 Torsion Angles for InT2CY_030723_0m.

A	B	C	D	Angle/°	A	B	C	D	Angle/°
S2	C3	C21	C4	175.9(3)	N20	C16	C9	C1	-178.3(3)
S2	C3	C21	C8	-4.0(4)	N20	C16	C9	C5	-0.7(4)
S2	C3	C	C11	2.6(3)	C1	C7	C19	O4	8.8(5)
S2	C15	C2	C13	-4.6(5)	C1	C7	C19	O	-168.8(5)
S2	C15	C11	C	0.2(4)	C1	C9	C5	C6	177.7(4)
O2	C14	C13	C2	-0.7(6)	C1	C9	C5	C8	-1.9(5)
O2	C14	C13	C18	-177.1(5)	C3	C	C11	C15	-1.9(5)
C21	C3	C	C11	-178.9(5)	C	C11	C15	C2	-176.9(4)
C21	C4	C10	C6	0.6(5)	C4	C10	C6	C5	-0.9(5)
C21	C8	C5	C6	-0.8(5)	O4	C19	C7	C17	-173.9(4)
C21	C8	C5	C9	178.7(4)	C2	C13	C14	O3	176.8(5)
N20	C6	C5	C8	-179.5(3)	O3	C14	C13	C18	0.4(6)
N20	C6	C5	C9	0.9(4)	C12	C1a	C1d	C1e	68.8(8)
N20	C6	C10	C4	179.7(5)	C1a	C1d	C1e	C1g	174.3(10)
N20	C12	C1a	C1d	176.7(6)					

Table 7 Hydrogen Atom Coordinates (Å×10^4) and Isotropic Displacement Parameters (Å^2×10^3) for InT2CY_030723_0m.

Atom	*x*	*y*	*z*	U(eq)
H8	2464(5)	2863(4)	4311(3)	71.0(13)
H10	392(6)	2073(5)	7037(3)	81.7(15)
H4	2847(6)	1504(5)	6646(3)	80.4(15)
H1	432(5)	4023(4)	3551(3)	78.2(14)
H	5233(5)	1022(5)	6294(3)	82.6(15)
H2	9200(5)	866(4)	4222(3)	81.2(15)
H11	7725(6)	659(5)	5605(3)	91.2(17)
H12a	-3387(6)	3077(6)	6575(3)	96.9(18)
H12b	-2039(6)	2284(6)	7006(3)	96.9(18)
H16	-2863(5)	4142(5)	5123(3)	78.6(15)
H1aa	-2422(6)	4387(6)	7548(3)	108(2)
H1ab	-3720(6)	5217(6)	7082(3)	108(2)
H1da	-4845(10)	5128(9)	8440(5)	166(3)
H1db	-5036(10)	4114(9)	7894(5)	166(3)
H1ea	-3262(15)	2243(11)	8353(8)	272(7)
H1eb	-2912(15)	3208(11)	8841(8)	272(7)
H1ga	-5470(90)	2810(120)	9324(15)	340(8)
H1gb	-4361(16)	2610(100)	9890(30)	340(8)
H1gc	-5490(90)	4120(20)	9700(40)	340(8)
H3	11105(4)	223(5)	1655(3)	162(2)
Ha	-2260(5)	5506(4)	1785(3)	155.2(19)

Table 8 Solvent masks information for InT2CY_030723_0m.

Number	X	Y	Z	Volume	Electron count	Content
1	0.113	-0.450	0.000	548.7	63.2	7.4 water

Electrochemical Properties

1. cyclic voltagramm

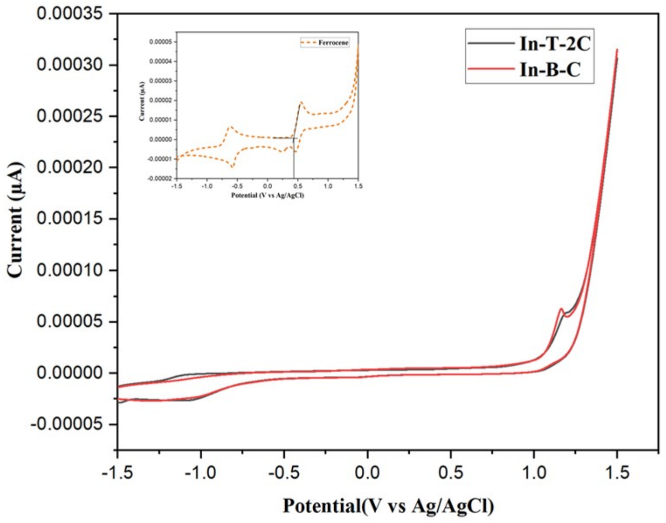

Theoretical studies data

1. Optimized structures of all novel indole-based compounds

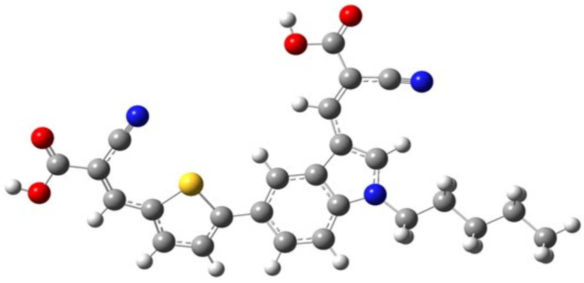

In-T-2C

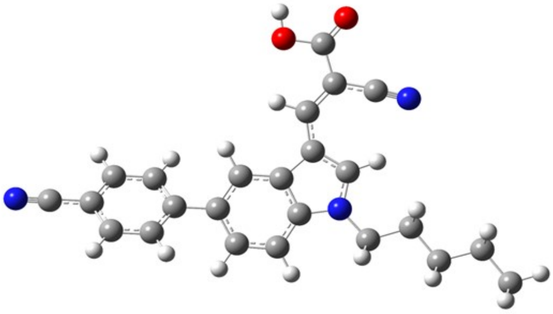

In-B-C

DSSC Fabrication

1. **Photoanodes**

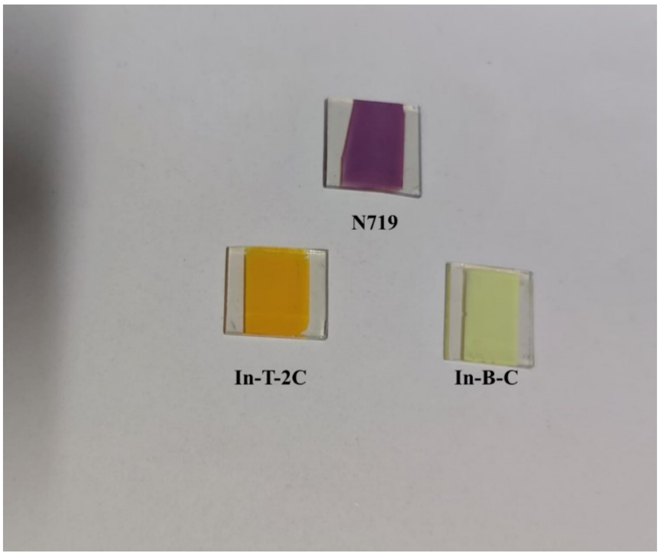

Compared compounds and their details

1. 1,6-bis[3-((E)-2-carboxyl-2-cyanovinyl)indol-1-yl] hexane

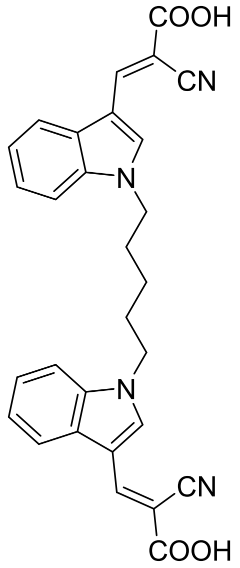

λ max = 379 nm

ε max (M^{-1} cm^{-1}) = 35,769

E_{LUMO} =-2.27 eV

η = 1.19 %

2. 2-cyano-3-(1-(10-hexyl-10H-phenothiazin-3-yl)-1H-indol-3-yl)acrylic acid

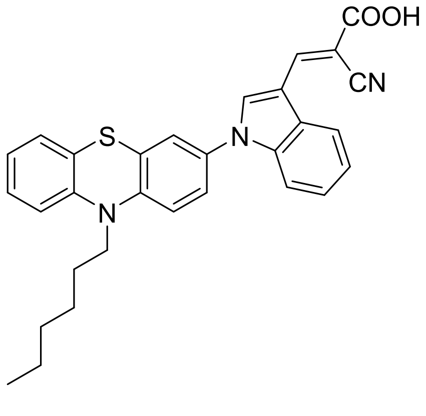

λ max = 379 nm

ε max (M^{-1} cm^{-1}) = 21,320

E_{LUMO} =-1.75 eV

η = 3.30 %

3. 4-((E)-(5-((Z)-1-cyano-2-(1-hexyl-1H-indol-3-yl)vinyl)thiophen-2-yl)methyleneamino)benzoic acid

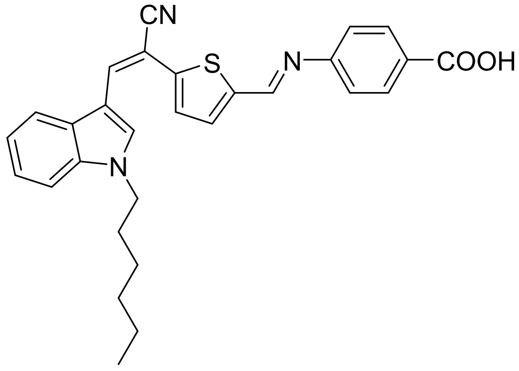

λ max = 452 nm

ε max (M^{-1} cm^{-1}) = 67,000

E_{LUMO} =-3.54 eV

η = 1.18 %

4. 3 (6 (2 Carboxy 2 cyanovinyl) 9 hexyl 9H carba zol 3 yl) 2 cyanoacrylic acid

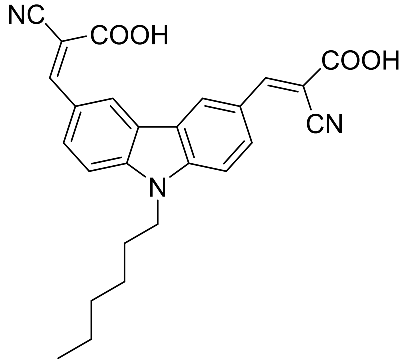

λ max = 395 nm

ε max (M^{-1} cm^{-1}) = 56,000

E_{LUMO} =-2.69 eV

η = 1.06 %

5. 6-(2-cyano-2-(5-(2,2-dicyanovinyl)thiophen-2-yl)vinyl)-9-(2-ethylhexyl)-9H-carbazole-3-carboxylic acid

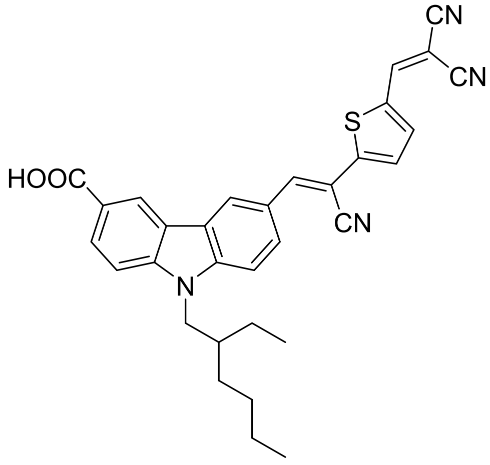

λ max = 404 nm

ε max (M^{-1} cm^{-1}) = 22,900

E_{LUMO} =-0.74 eV

η = 0.0027 %
